# Parametric nasopharyngeal swab for sampling COVID-19 and other respiratory viruses: Open source design, SLA 3-D printing and UV curing system

**DOI:** 10.1016/j.ohx.2020.e00135

**Published:** 2020-08-29

**Authors:** Nicole Gallup, Adam M. Pringle, Shane Oberloier, Nagendra G. Tanikella, Joshua M. Pearce

**Affiliations:** a Department of Biomedical Engineering and Mechanical Engineering, Michigan Technological University, Houghton, MI 49931, USA; b Department of Materials Science & Engineering, Michigan Technological University, USA; c Department of Electrical & Computer Engineering, Michigan Technological University, USA; d Équipe de Recherche sur les Processus Innovatifs (ERPI), Université de Lorraine, France; e School of Electrical Engineering, Aalto University, Finland

**Keywords:** Open hardware, COVID-19, Medical hardware, Nasopharyngeal swab, Nasal swab, UV curing, SLA, RepRap, 3-D printing, Additive manufacturing

## Abstract

Access to nasopharyngeal swabs for sampling remain a bottleneck in some regions for COVID-19 testing. This study develops a distributed manufacturing solution using only an open source manufacturing tool chain consisting of two types of open source 3-D printing and batch UV curing, and provides a parametric fully free design of a nasopharyngeal swab. The swab was designed using parametric OpenSCAD in two components (a head with engineered break point and various handles), which has several advantages: i) minimizing print time on relatively slow SLA printers, ii) enabling the use of smaller print volume open source SLA printers, iii) reducing the amount of relatively expensive UV resin, and iv) enabling production of handle on more accessible material extrusion 3-D printers. A modular open source UV LED box was designed, fabricated for $45 and tested for batch curing. Swabs can be fabricated for $0.06-$0.12/swab. The results of the mechanical validation tests showed that the swabs could withstand greater forces than would be expected in normal clinical use. The swabs were also able to absorb a significant amounts of synthetic mucus materials and passed abrasion and handling tests. The results show the open source swab are promising candidates for clinical trials.

## Graphical abstract

**Figure:**
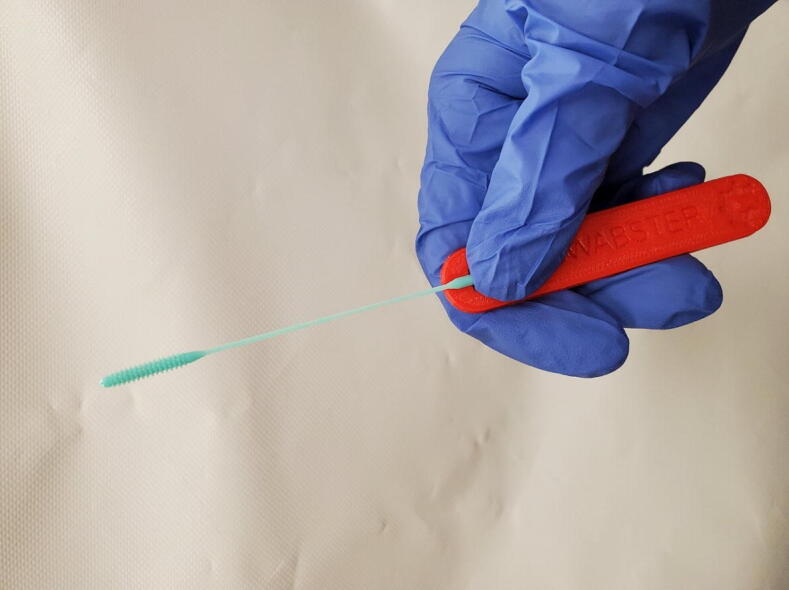

## 1 Specifications table

**Table:** 

Hardware name	Open Source Nasopharyngeal Swab Open Source Modular UV Curing System	
Subject area	• Medical	
Hardware type	• Medical sample handling and preparation	
Open Source License	GNU General Public License (GPL) v3.0 and CERN Open Hardware License (OHL) v1.2	
Cost of Hardware	Nasopharyngeal Swabs:	$0.060/swab with press-fit handle
		$0.114/swab for tong style handle
	UV Curing System: $45.42	
Source File Repository	https://osf.io/z5jgu/ registration: https://osf.io/yax7q	

## 2 Hardware in context

Coronavirus disease 2019 (COVID-19) caused by SARS-CoV-2 is increasing mortality rates by overwhelming medical infrastructure at the regional level [1], [2], [3], [4]. There are critical shortages of ventilators [5], [6], [7], personal protective equipment (PPE) [8], [9], [10], [11], [12], and testing [13], [14] including swabs [15]. The latter is in such short supply coroners are concerned that the test shortages are leading to severe undercounting of COVID-19 tests in the U.S. [16]. Manufacturers throughout the world have ramped up production when possible and the White House has recognized that SARS-CoV-2 represents a national emergency and has implemented the Defense Production Act (DPA) [17]. One of the products the DPA has been applied to is swabs to compel an unnamed company to produce 20 million more coronavirus testing swabs per month [18]. This centralized top down approach where one large company is forced to manufacture something may provide a solution to critical medical hardware shortages. Another approach, however, is to provide products uses a distributed approach. In this approach, technically and economically-viable open source small-scale digital technologies are used for distributed manufacturing [19], [20] using small-businesses [21], fab labs [22], [23], or even individual home-based production [24], [25], [26], [27]. This open source hardware design approach [28], [29], [30] has been applied to medical equipment [31], [32], [33], [34] and has been shown to be particularly adept at overcoming supply shortages [35], [36], [37], [38], [39].

The U.S. remains well below the rest of the developed world in COVID-19 testing, while suffering the most deaths of any nation and there is a clear case for mass testing and contact tracing to end the pandemic [40], [41], [42], [43]. The three legs of the stool of testing are labs/personnel, media, and nasopharyngeal swabs. This study develops a distributed manufacturing solution using an open source manufacturing tool chain to the third requirement: the nasopharyngeal swab.

The U.S. CDC, recommends that testing for COVID-19 on nasopharyngeal swabs made from synthetic fiber swabs with plastic shafts are used as calcium alginate swabs or swabs with wooden shafts may contain substances that inactivate some viruses and inhibit PCR testing [44]. Thus, the materials used for swab and handle are UV-curable polymer resin and thermoplastic as the former has been tested [45] and the latter does not contact the patient. Many companies and research groups have developed different kinds of swabs [45], [46]. This in itself creates a problem for regulators because of the wide array of designs, materials and printers, many of which are trying to corner specific intellectual property. Callahan et al. have evaluated 160 of swab designs and validated four designs [45]. In addition, a 3-D printed swab consortium has formed [46]. 3-D printed nasopharyngeal swabs have been commercialized from the consortium for proprietary printers from Carbon, FormLabs, Envisiontec, Origin, and Abiogenix. All of the consortium’s manufacturers have been verified as ISO13485 facilities and are in production of ‘FDA registered’ swab designs. The 3-D printer manufacturers have tested and verified the swab designs on their own printers. These designs can be used on other similar printers, but then must be retested and reverified. For example, the company FormLabs has worked with several partners to relatively-openly design and test various materials and geometries of swabs including Beth Israel Deaconess Medical Center [47], University of Southern Florida [48], the University of Washington, Stanford University, MIT and the United States Army [49]. Working collaboratively, they have developed test swabs to be printed on high-resolution stereolithography (SLA) 3-D printers and are now being used [50].

## 3 Hardware description

As FormLabs printers (and other major SLA 3-D printer manufacturers) are deployed throughout the world they offer a method for distributed manufacturing of nasopharyngeal swabs to help relieve the swab shortage. FormLabs and the other printers in the consortium, however, are proprietary and costly, with a full system costing $3,500-$10,000 for FormLabs as an example [51]. In addition, the swab designs themselves are propriety. For example, the Northwell Swab, that is freely shared now comes with a warning: “This 3D print swab design protocol is patent protected. The University of South Florida grants the recipient of design files permission to 3D print and use the swabs for non-commercial purposes until April 15, 2021.” [52]. This may cause deployment concerns based on potential liability in the future. In addition, the source, originally available in ref. [52] along with the notice was removed by July 28, 2020.

**Fig. 1:**
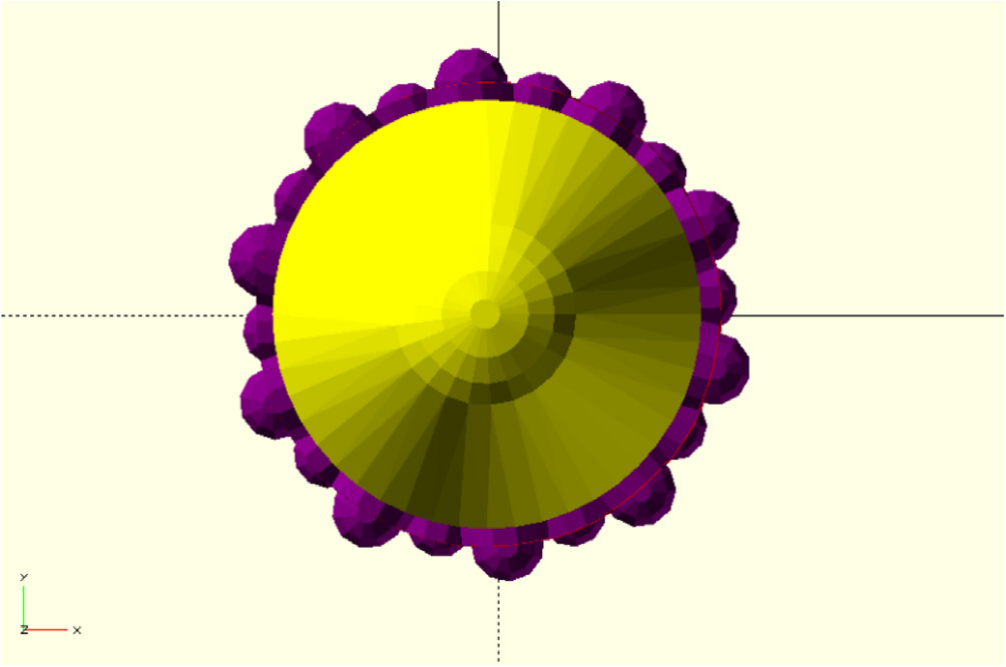
Top view of rounded top of nasal swab in yellow with hemisphere tip of adjustable radius, protuberance, and roundness with purple nobs surrounding in offset sequence.
The front and rear of the swab head are tapered to enable easy insertion and extraction of the swab as seen in Fig. 2. The spacing between nobs is selected both for printability and ensuring space for specimen collection.

To overcome these limitations, this study develops a distributed manufacturing solution using only an open source manufacturing tool chain and provides a parametric fully free design of a nasopharyngeal swab. Following well-established open hardware design protocols [28], [29], [30], the swab was designed using parametric OpenSCAD [53]. This approach has several advantages over the bench mark designs:

The front is rounded to reduce the likelihood of injury in the patient’s nasal cavity as seen in Fig. 1. The nobs are positioned in an offset sequence so that each row is rotated one nob thickness in the X-Y plane. This is to ensure ideal specimen collection and retention on swab while moving in the nasal cavity.

**Fig. 2:**
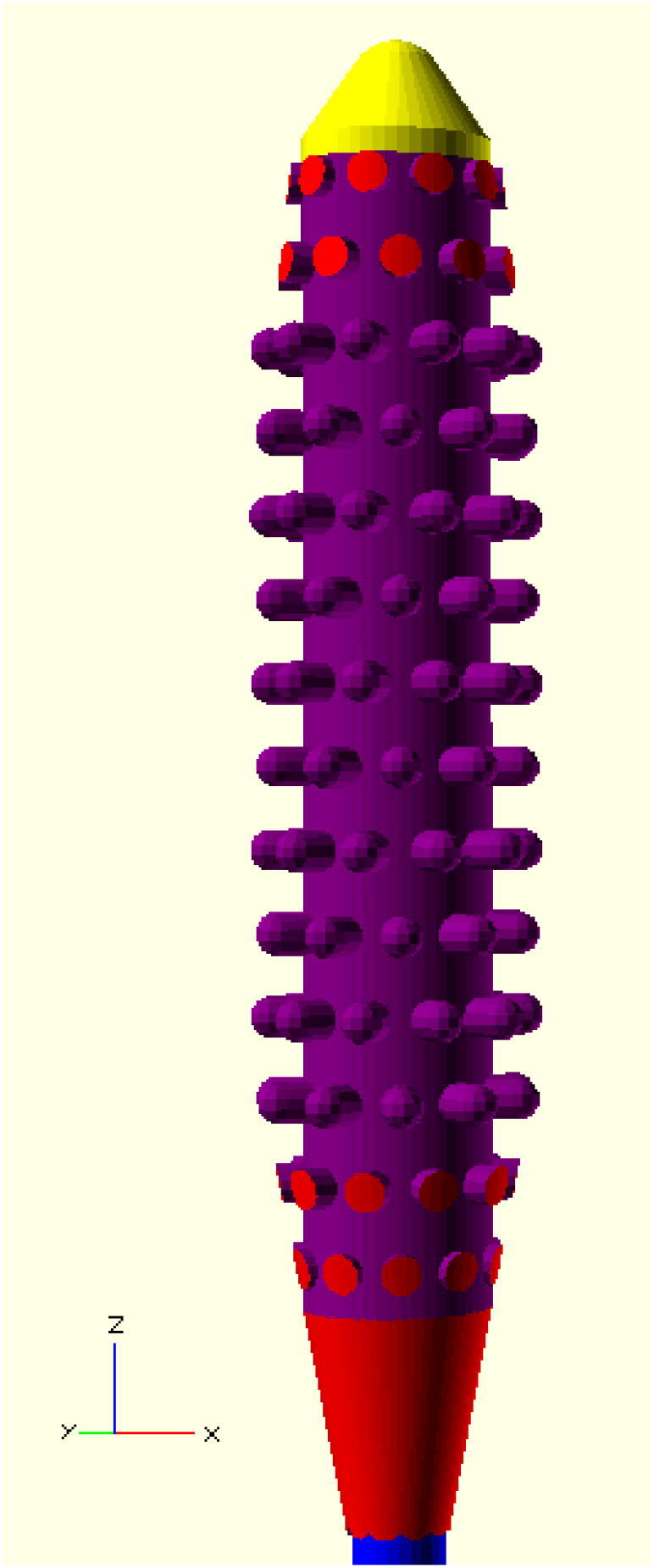
Side view of head of tip of open source swab with top in yellow, tip and nobs (arms) in purple, tapered sections in red, and neck in blue.
The swab is shortened and fit with a handle. This has several advantages including: i) minimizing print time on relatively slow SLA printers, which increases throughput for any SLA printer, ii) enabling SLA printers with smaller print volumes (like the open source and relatively less costly Prusa SL1 [54]) to print the swab, iii) reduces the amount of relatively expensive UV resin, and iv) pushes production of the handle over to the more numerous, accessible and faster fused filament fabrication (FFF) over a wide variety of commercial filaments with acceptable mechanical properties [55] or other material extrusion based printers such as fused particle fabrication (FPF) and fused granule fabrication (FGF) [56]. This latter point is perhaps the most important because SLA-based printers are far less numerous than FFF-based printers. Using the two-part design cuts the SLA print time down and allows it to print on small SLA printers, while enabling production rates to be held up with more numerous FFF-based printers used in parallel.
The design is fully parametric so any feature can be altered in the future for a specific test, patient, disease or circumstance. For example, as shown in Fig. 3, the handle can be customized with existing test to for example brand it with a specific hospital, State, funder, or company providing the test. Another example is to use different types of handle (see tong handle in Fig. 4). The press-fit handle minimizes material cost and print time, however, requires pressing the swab base into the handle manually. The tong handle is more expensive in material and print time, however, it distances the user from the swab throughout the entire process. Depending on the tolerances of a specific FFF/FGF/FPF machine the press fit in the handle can be adjusted in the OpenSCAD to work with the SLA printed swab to enable the fit function to work.

**Fig. 3:**
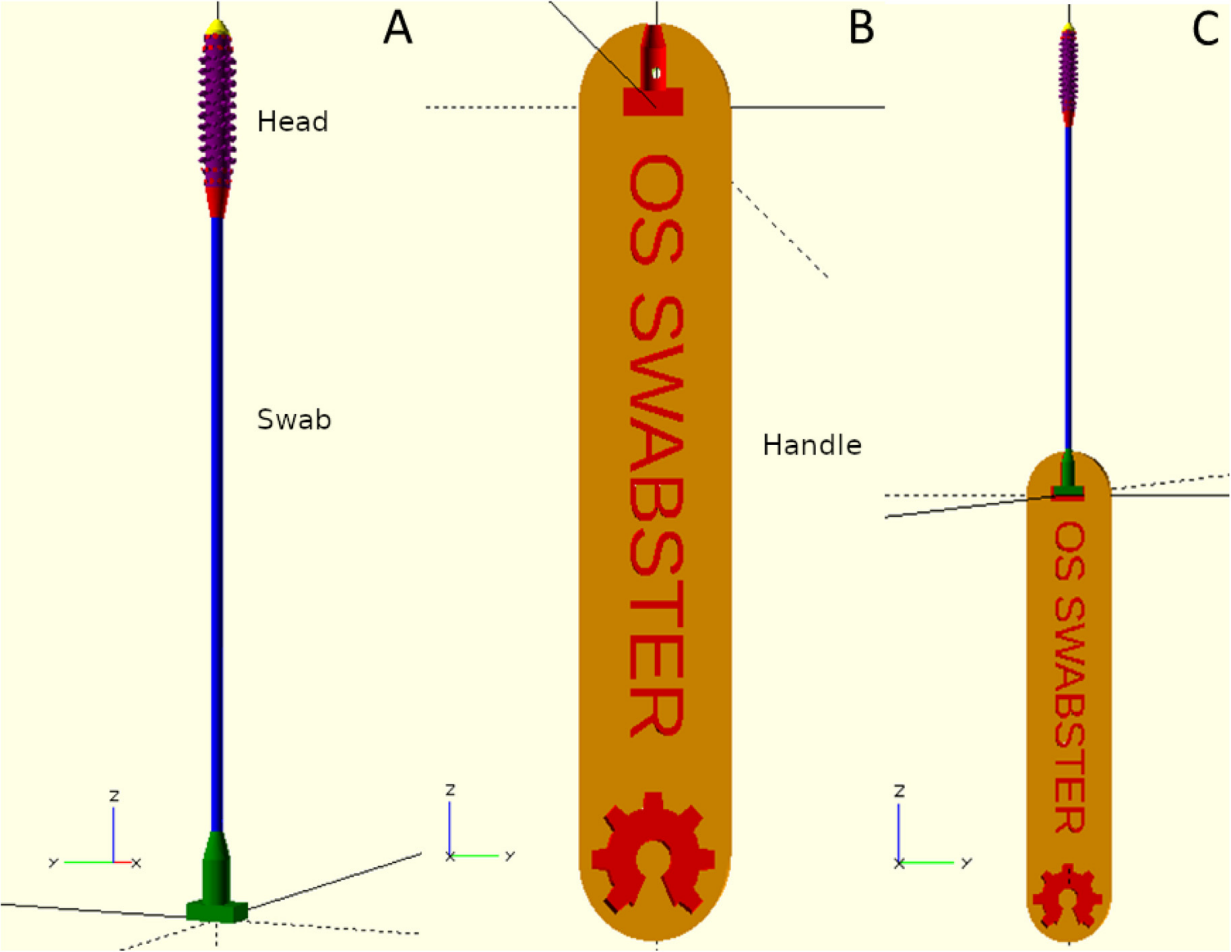
OpenSCAD rendering of A) full side view of swab; B) press-fit handle with customizable text; and C) swab and handle assembled.

**Fig. 4:**
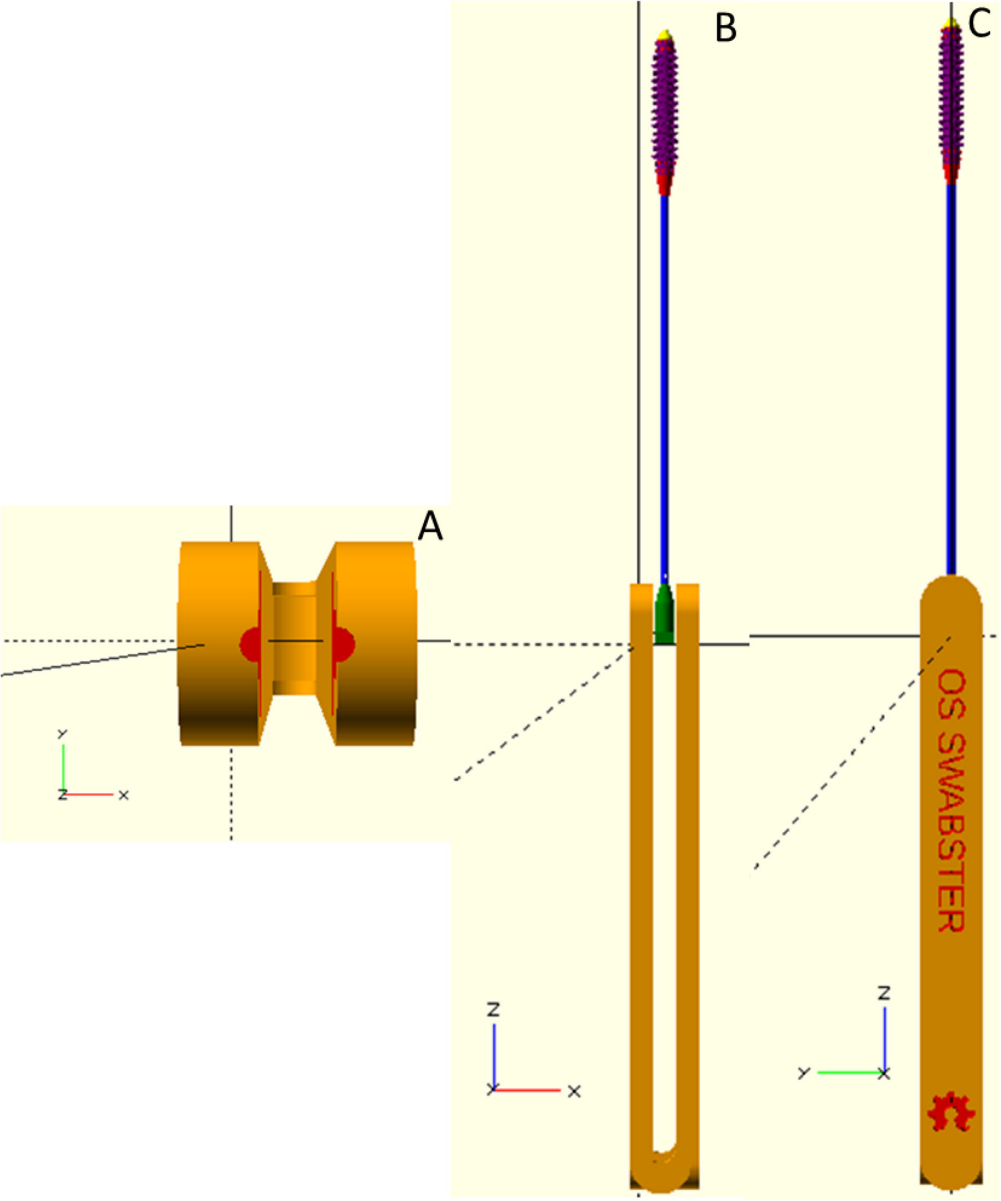
OpenSCAD rendering of A) top (lengthwise) view of clamping section of tong handle; B) tong handle showing swab placement in relaxed state for easy removal (side view); and C) swab and handle assembled in front view profile with customizable writing.
Mechanical testing on patented 3-D printable swabs showed that they often broke in the neck of the swab when under substantial loads because there was no engineered weak point in the swab. This creates a risk that the swab may break in the patient’s nose causing harm and/or being difficult to remove. To ensure that the shaft snaps off in the same location outside of the patient’s nose the swab is engineered to fail at a specific location. This is accomplished with a small hole, which can be adjusted in position and radius, located near the transition point from the lower body of the swab to the base section to be put into the handle. The hole is parallel to the press fit bar (see Fig. 5) so it provides a clear direction to press the body of the nasal swab for it to break consistently. At this point the swab has the least material present per unit cross section and is thus the weakest. The finalized assembled swab is shown in Fig. 6.

**Fig. 5:**
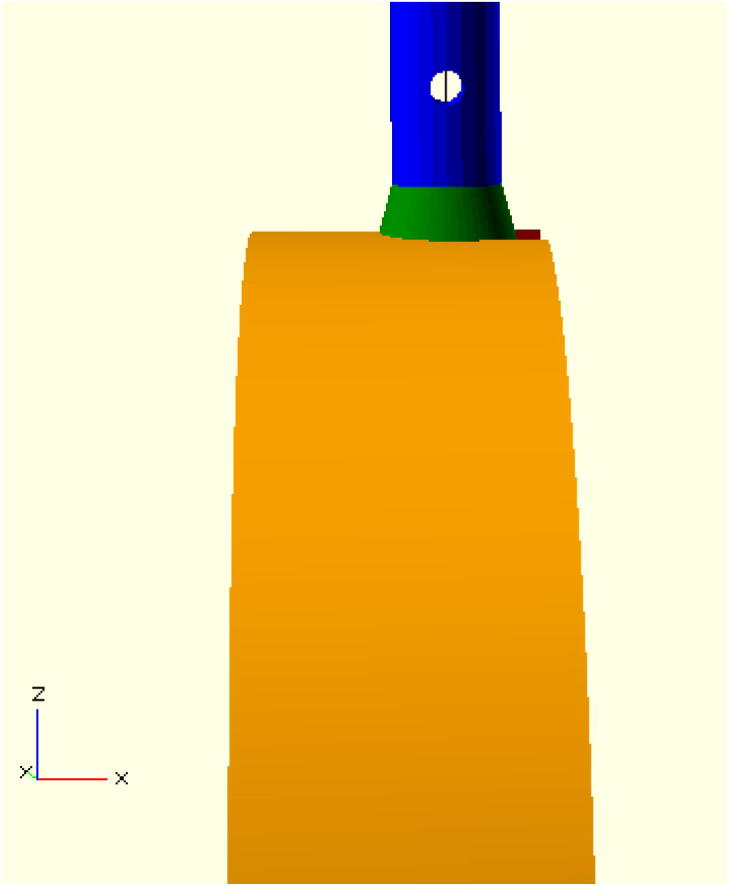
Detail of breakaway hole with neck of swab in blue, base in green, and handle in orange.

**Fig. 6:**
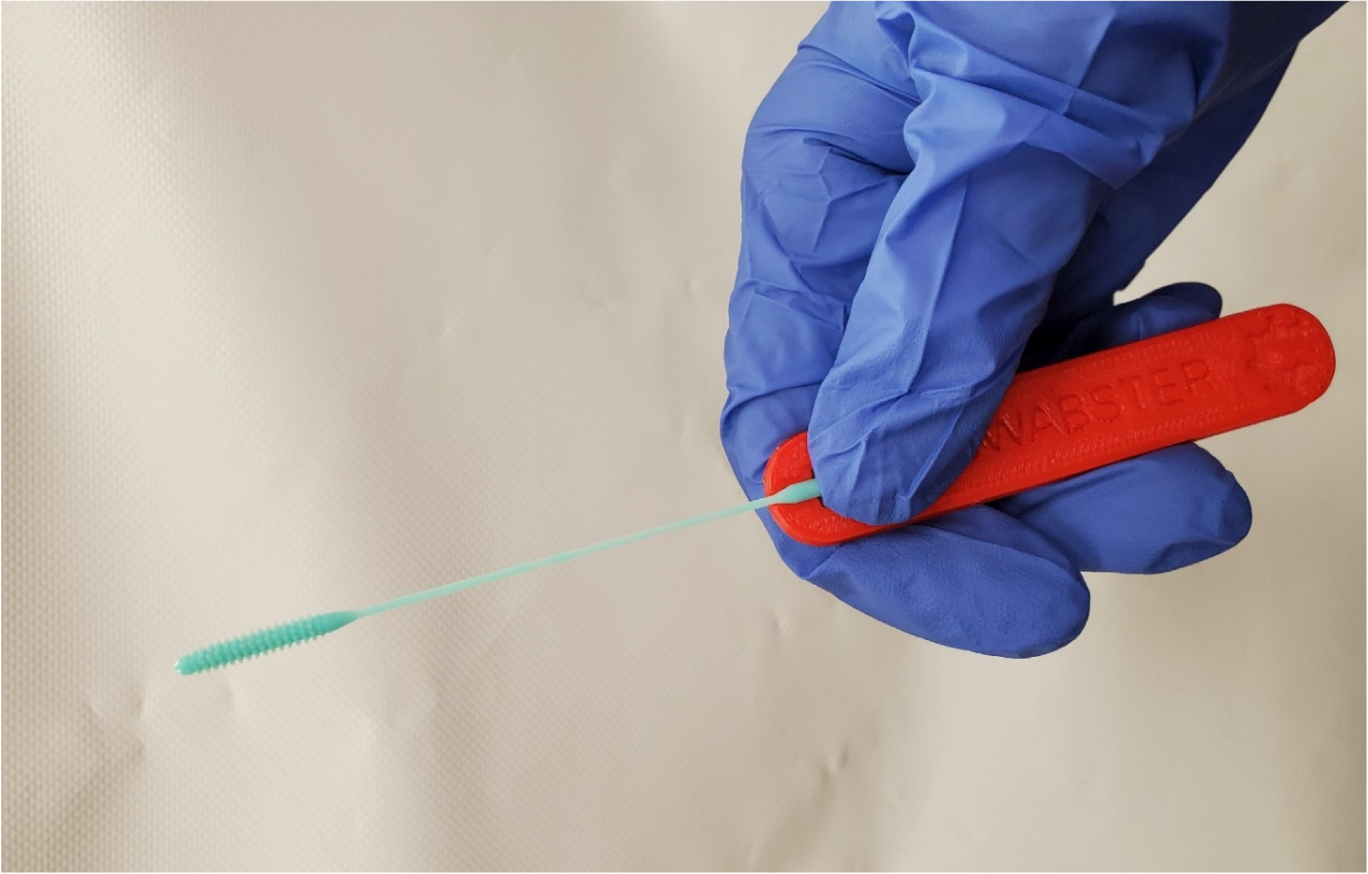
Assembled parametric swab and handle.

These design features allow the device to be fabricated on open source 3-D printers developed as part of the RepRap project [57], [58], [59]. The swab tip is 3-D printed on an open source Prusa SL-1 [54] and the handle on an open source Lulzbot Taz 6 [60]. To further cure the resin print head, an open source UV LED box was designed, fabricated and tested. This UV curing box, which is constructed from PCBs, is itself modular so it can be used for UV illumination of other applications.

• Swabs can be fabricated 9 min./swab/SLA printer at a cost of $0.014 for the swab head alone and $0.0458 for the swab handle on a FFF machine for a total cost of $0.0603/swab, but perhaps more importantly can be done following a distributed manufacturing paradigm. For context, the traditional manufacturing of a patented single swab is $0.35 with transport medium [61].
• The swab design is two components, both of which can be 3-D printed, although it allows for the slow AM process to be minimized and can be used on small volume SLA printers.
• The open source nasal swab design is written in parametric OpenSCAD so other users can personalize it and customize it for their application (e.g. infant/child swabs)
• The UV curing system is also parametric and modular enabling the system to be used for any type of UV curing. The LEDs can be swapped out for different wavelengths to be used for other applications (e.g. higher energy UV diodes could be used for virus sterilization).

## 4 Design files

### 4.1 UV curing system

#### 4.1.1 UV curing system design files summary

**Table:** 

Design file name	File type	Open source license	Location of the file
*/Gerbers*	*GBR, DRL*	GPL3.0	https://osf.io/z5jgu/
*Brackets.scad*	SCAD	GPL3.0	https://osf.io/z5jgu/
*Corner.stl*	STL	GPL3.0	https://osf.io/z5jgu/
Edge.stl	STL	GPL3.0	https://osf.io/z5jgu/
Plate.stl	STL	GPL3.0	https://osf.io/z5jgu/
UV_Curing_Box-cache.lib	lib	GPL3.0	https://osf.io/z5jgu/
UV_Curing_Box.kicad_pcb	KICAD_PCB	GPL3.0	https://osf.io/z5jgu/
UV_Curing_Box.net	NET	GPL3.0	https://osf.io/z5jgu/
UV_Curing_Box.pro	PRO	GPL3.0	https://osf.io/z5jgu/
UV_Curing_Box.sch	SCH	GPL3.0	https://osf.io/z5jgu/

1. The files available in the /Gerbers directory are composite files generated from the PCB design process and are used for ordering copies of the boards from fabrication shops.
2. Brackets.scad is the editable source file for all of the 3-D printed components of the curing system.
3. Corner.stl, Edge.stl, and Plate.stl are the outputs generated from the SCAD file and are ready for immediate slicing and printing on any RepRap class 3-D printer.
4. UV_Curing_Box-cache.lib hosts footprints and symbols used in the schematic and board design. UV_Curing_Box.kicad_pcb is the physical board design.
5. UV_Curing_Box.net is a list of components and connections which is used when transferring from a schematic design to a PCB design.
6. UV_Curing_Box.pro is the project file for the design (used for logistical purposes).
7. UV_Curing_Box.sch is the schematic file for the design.

### 4.2 Nasopharyngeal swab design files

#### 4.2.1 Nasopharyngeal swab design files summary

**Table:** 

Design file name	File type	Open source license	Location of the file
*OS_nasal_swab.stl*	STL	GNU General Public License (GPL) v3.0	https://osf.io/z5jgu/
OS_swab_handle.stl	STL	GNU General Public License (GPL) v3.0	https://osf.io/z5jgu/
OS_tong_handle.stl	STL	GNU General Public License (GPL) v3.0	https://osf.io/z5jgu/
OS_nasal_swab_and_handle.scad	SCAD	GNU General Public License (GPL) v3.0	https://osf.io/z5jgu/

The OS_nasal_swab.stl file is the printable file for SLA printers. No supports should be used, and the rectangular base should be flat on the build plate.
The OS_swab_handle.stl file is the printable file for FFF printers. It should lay flat, with the subtracted text and OSHW logo facing up.
The OS_tong_handle.stl is an alternative handle printable file for FFF printers. For printers that are not optimally calibrated this may be a better choice as the hold on the swab is more secure.
The OS_Nasal_swab_and_handle.scad file is the master file for altering either the swab or handle. To us, render the correct part, and alter the variables to change features as desired.

## 5 Bill of materials

### 5.1 UV curing system Bill of materials

**Table:** 

Designator	Component	Number	Cost per unit-currency	Total cost-currency	Source of materials	Material type
*Circuit Board*	UVLED Board	12	$2.10	$25.20	In House	NA
*Bolt*	M3 × 10 mm bolt	48	$0.07	$3.16	Amazon	NA
*Wire*	18 AWG wire	1 m	$0.72 per m	$0.72	Amazon	NA
*Power Supply*	12VDC 5A Supply	1	$11.99	$11.99	Amazon	NA
*Power Cable*	IEC Power Cord	1	$3.99	$3.99	Amazon	NA
*Edge*	Edge Bracket	12	$0.02	$0.24	In House	PLA
*Corner*	Corner Bracket	8	$0.02	$0.08	In House	PLA
*Plane*	Face Bracket	2	$0.02	$0.04	In House	PLA

Many of the components in this assembly have multiple (and potentially less expensive) sources. The PLA components costs are assuming a $22.99 / kg spool.

### 5.2 UV LED Bill of materials

**Table:** 

Designator	Component	Number	Cost per unit-currency	Total cost-currency	Source of materials	Material type
*Circuit Board*	PCB	1	$0.57	$0.57	JLCPCB	NA
*LED*	UV LED	12	$0.07	$0.83	Amazon	NA
*Conn*	Screw Terminals	4	$0.17	$0.70	Amazon	NA

The UV LED type can vary as needed, but for this application 395 nm UV LED’s are used [62]. The LED choice is only constrained by the 2.54 mm through-hole footprint.

### 5.3 Nasopharyngeal swab Bill of materials

**Table:** 

Designator	Component	Number	Total cost-currency	Source of materials	Material type
*Swab*	Swab UV resin	1	$0.0145	Prusa	UV resin
*Swab handle*	Handle plastic	1	$0.0458	Lulzbot	PLA/PETG/ABS
*Swab Tongs*	Handle Plastic	1	$0.0993	Lulzbot	PLA/PETG/ABS

The components were massed on a calibrated open source digitally lab-grade scale [63]. The masses were 0.25, 1.91 and 4.14 g for the swab, handle and tongs, respectively. The cost of the components was calculated by mass × (cost per unit mass) the cost of the resin is $57.99/kg and the cost of the filament is $23.99/kg. The resin used here is made up of epoxy resin (CAS 61788–87-4, which is a phenolic epoxy resin 40–50%), a monomer (CAS 13048–33-4 which is 1,6-hexanediol diacrylate 20–40%) and photoinitators (CAS 947–19-3, which is 1-hydroxycyclohexyl phenyl ketone 3–5%). In addition, resins from Prusa Polymers have a 2–5% color pigment. The swab head is made from the Prusa UV sensitive 3-D printing tough resin and can be purchased in 1-kg bottles online. The swab handle and tongs are both made form Hatchbox PLA filament as tested and printed on a Lulzbot Taz 6. The overall cost of a full swab is $0.0603 with press-fit handle and $0.1138 for the tong style handle.

## 6 Build instructions

### 6.1 UV curing system fabrication

The UV curing system is parametric in that it consists of modular components which can be linked together to create any size cubic container. As a first step, users can determine the geometry of the curing system with each edge being divisible by 4 in. (since the board has an edge diameter of 4 in.). For this implementation the box is 4″x8″x8″. From the dimensions the number of components need can be determined – in this implementation there are 12 boards required.

**Fig. 7:**
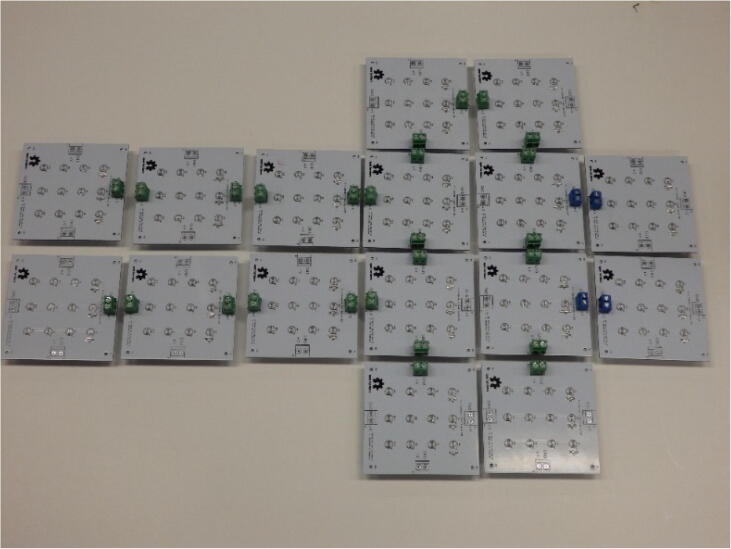
The UV curing box is first laid out in order to determine terminal block placement.

It is not necessary to solder all screw terminals onto the boards, as they are not all necessary depending on the configuration. To determine where to place the connectors, the boards are laid out in their “unfolded” state, as seen in Fig. 7, and terminal block pairs are places such that power can be supplied to each board at least once. Redundant connections will not hurt the system.

The boards have provisions for resistors if it is determined that the light intensity needs decreased, but for this application max intensity is desired. In this case, 0 Ohm resistors can be soldered in place, or to cut costs further, the clippings from the UV LEDs can be used to bridge the two pads. The UV LED soldering requires no special treatment – aside from the fact that LEDs are polar and therefore must be soldered in the proper orientation. The LEDs should be soldered on the opposite side of the silkscreen such that they will be facing the interior of the box.

**Fig. 8:**
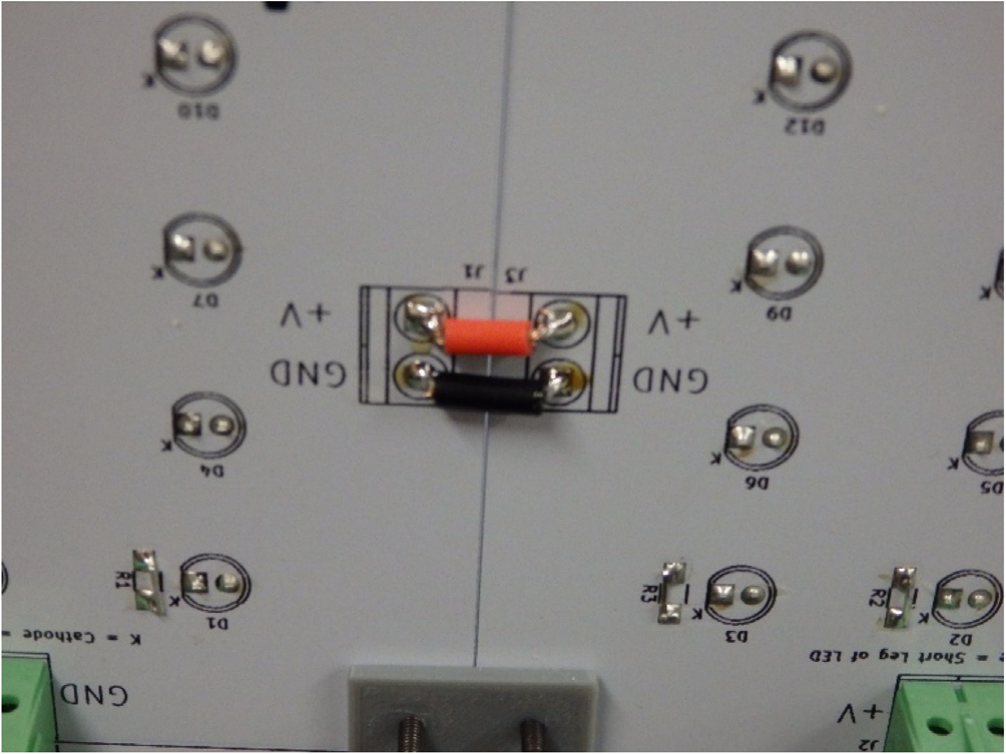
Wires directly soldered to transfer power from one board to the next.

If cost is a major concern, the terminal blocks can be replaced with directly soldered wire as seen in Fig. 8. This is undesirable in most cases as it reduces the maintainability of the system (i.e. it cannot be as easily disassembled as it would need to be de-soldered).

**Fig. 9:**
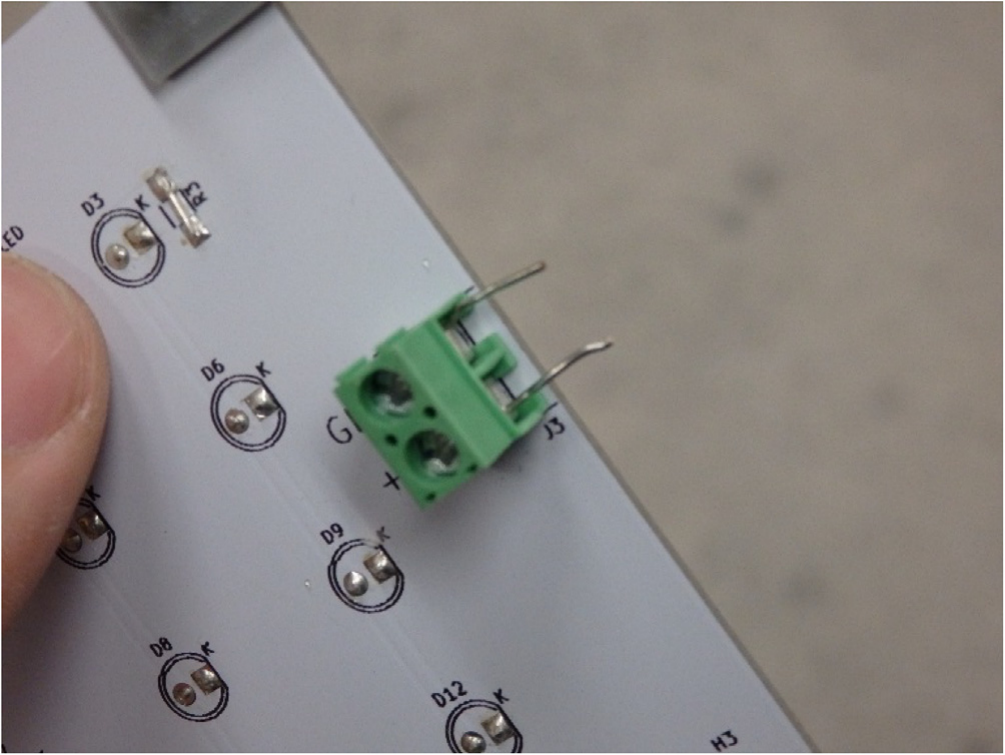
Clipped LED leads placed into screw terminals. The other ends will get clamped in adjacent connector.

Once the boards have been fully soldered, the next step is to identify boards that are on the same plane – specifically terminal blocks that are intended to be connected to one another. Since they are butted up end to end, placing typical wire in the connectors is not an option. Instead, solid resistor leads are cut to the correct length and clamped in place (Fig. 9).

At this point, the necessary brackets can be printed, and the boards can be bound together. The M3 bolts can be applied two ways – they can either be fed through the exterior of the box and then terminated with a nut, or in the case of this application, the bolts are fed through the interior and thread directly into the plastic. No nuts are necessary.

**Fig. 10:**
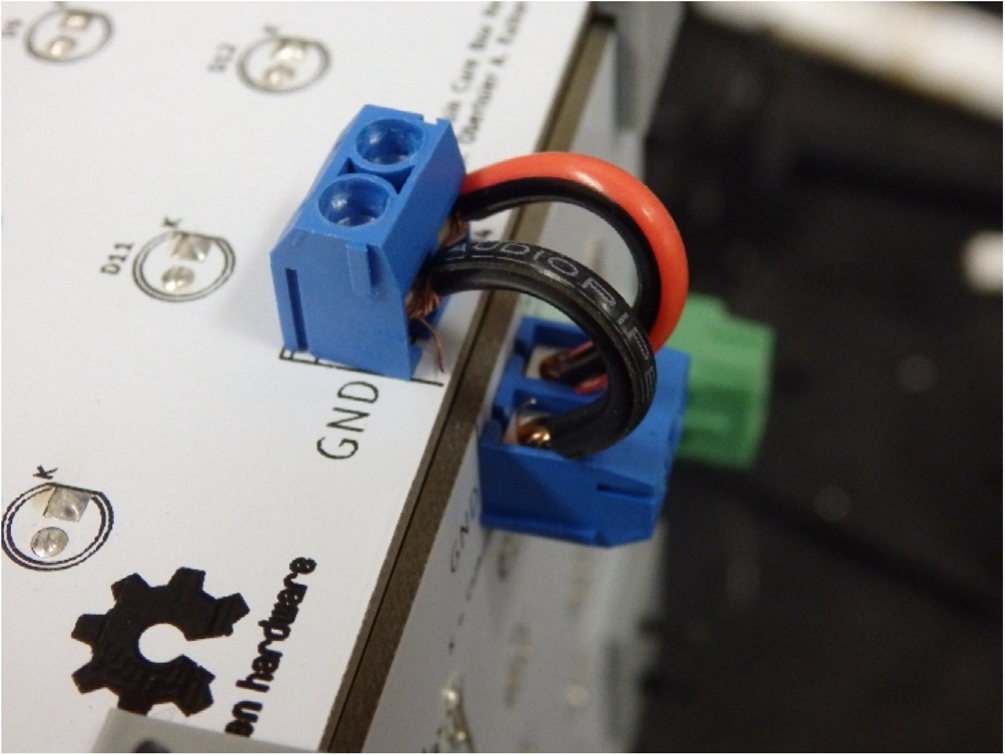
Wires connecting two terminal blocks on a corner. The wires are made longer than necessary as they double as the hinge for the lid.

Next, the corner joints can be wired using any standard insulated wire (Fig. 10).

**Fig. 11:**
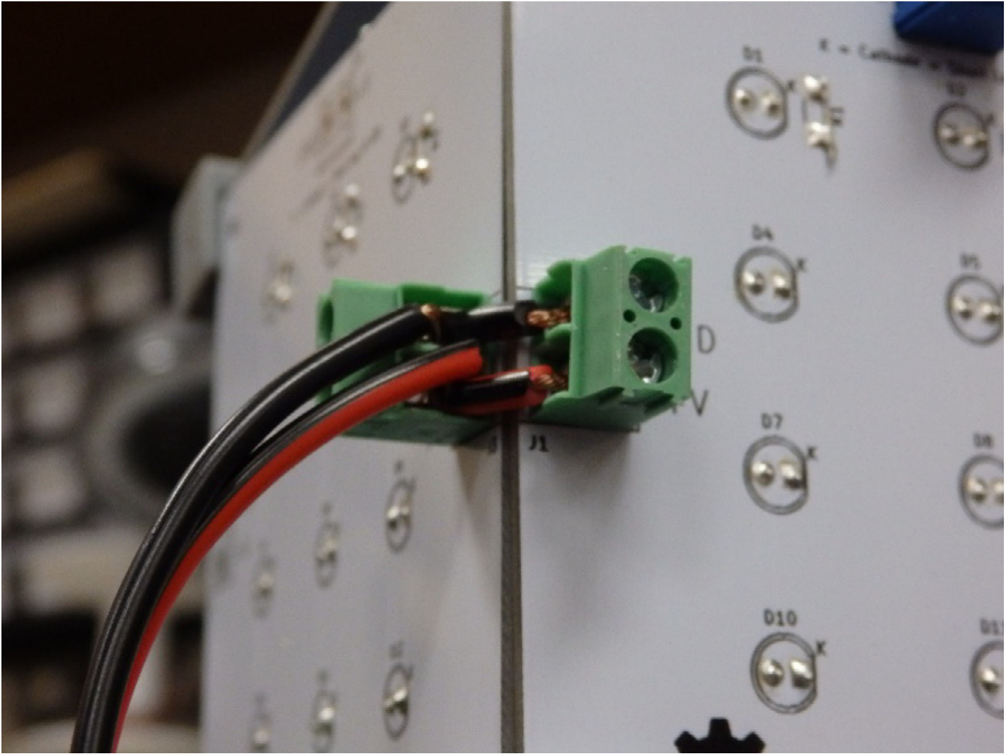
Two cables tapped into a terminal set for power flow from the supply.

Two lengths of wire can also be tapped into one of the screw terminal sets (Fig. 11) and wired to their respective plus and minus terminals on the power supply. The IEC power cable can have its end cut and stripped. Though the color key may vary, typically the green wire will connect to the ground terminal of the supply, the lightest color will tie to the neutral terminal, and the darkest color will tie to the live terminal. This wiring color scheme should be verified on a case by case basis.

**Fig. 12:**
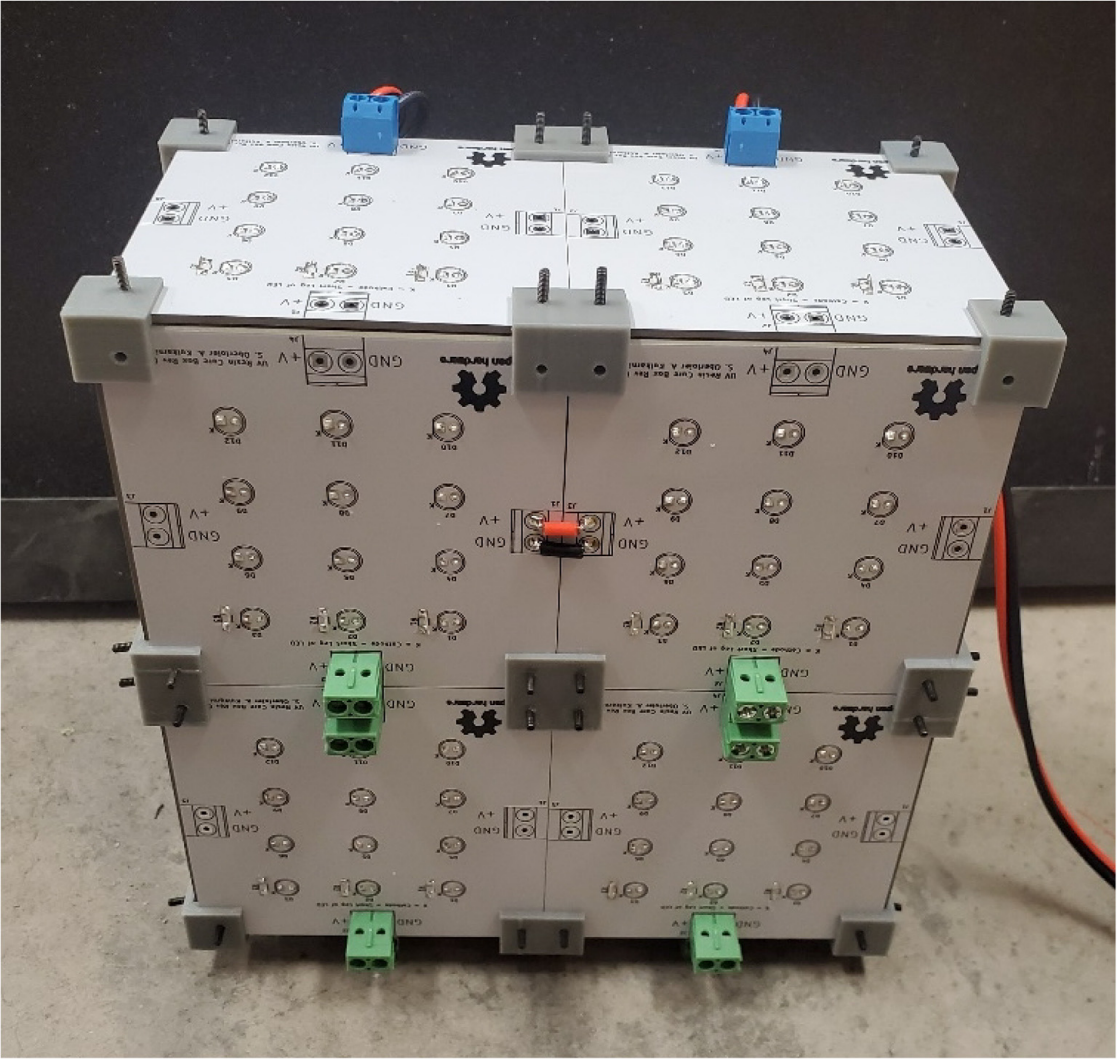
The exterior of the completed open source UV curing box.

Before powering on the completed box (Fig. 12), a multimeter should be used to measure the input resistance of the box, if it is a low number (around 5 or less Ohms), there is likely a short.

**Fig. 13:**
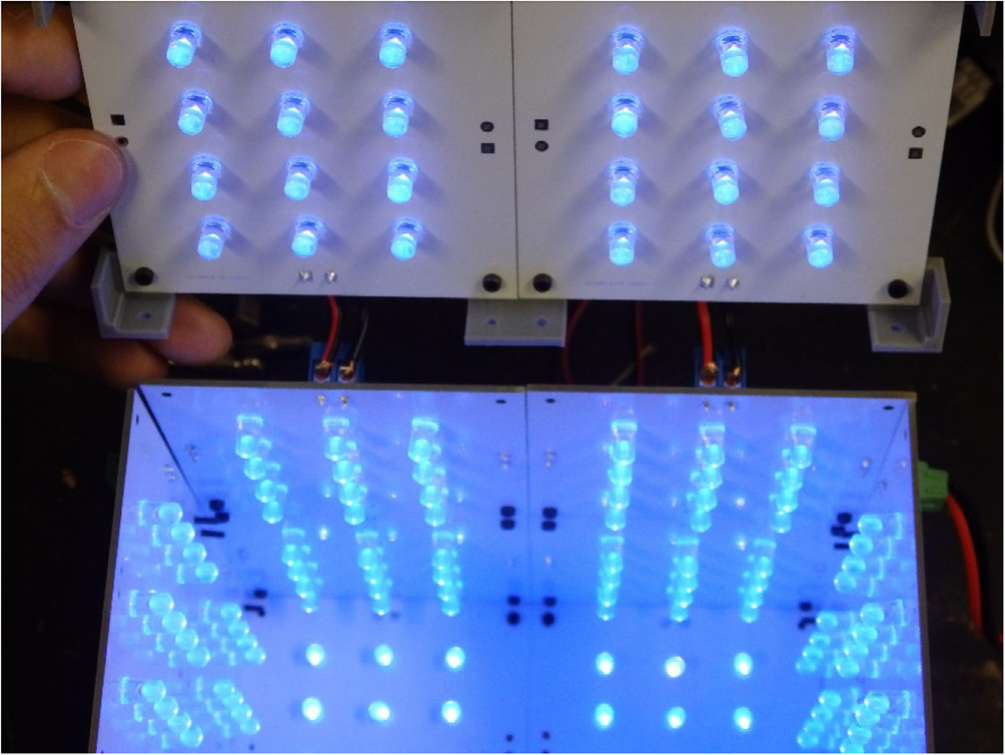
Inside of the powered-on curing box. Note the wires being used as hinges and the corner and edge brackets are fully extended so the top can correctly align when it is placed.

Fabricators should double check each connection point and verify there are no loose strands of wire. Once the box is short free, it can be plugged in, and its functionality can be verified. It is important to point out that the LEDs are directional and will seem dimmer when viewed from the side (Fig. 13).

**Fig. 14:**
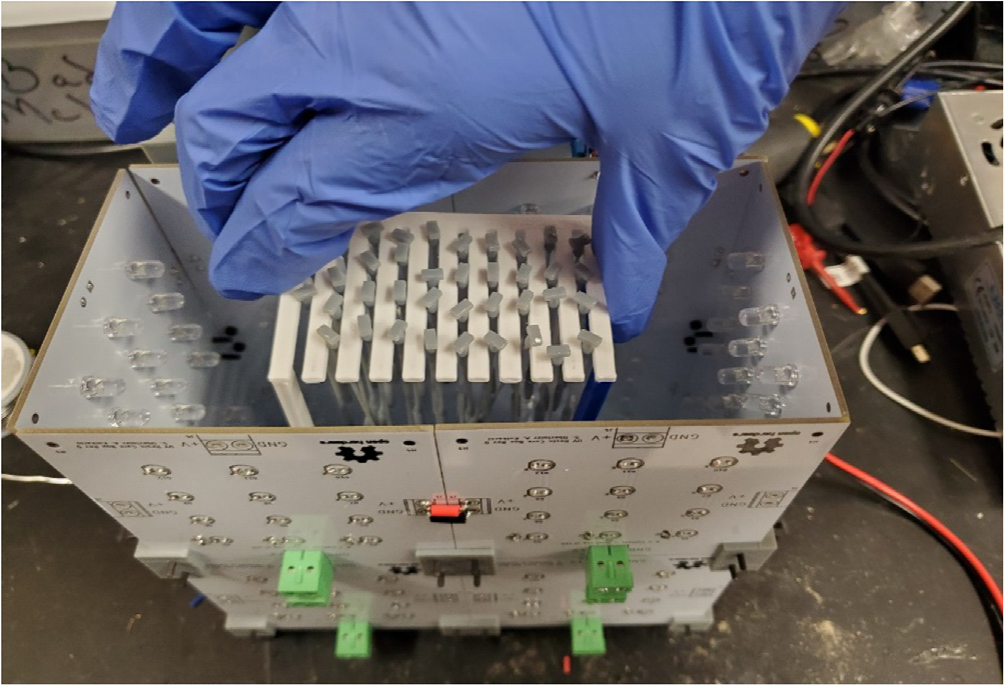
Inserting swabs into UV curing box.

Swabs are placed in a 3-D printed holder made up of four legs (https://osf.io/m5hqf/) and one top (https://osf.io/yzfa2/) by hanging them upside down and then placed in the UV curing box (Fig. 14).

### 6.2 Swab fabrication

**Fig. 15:**
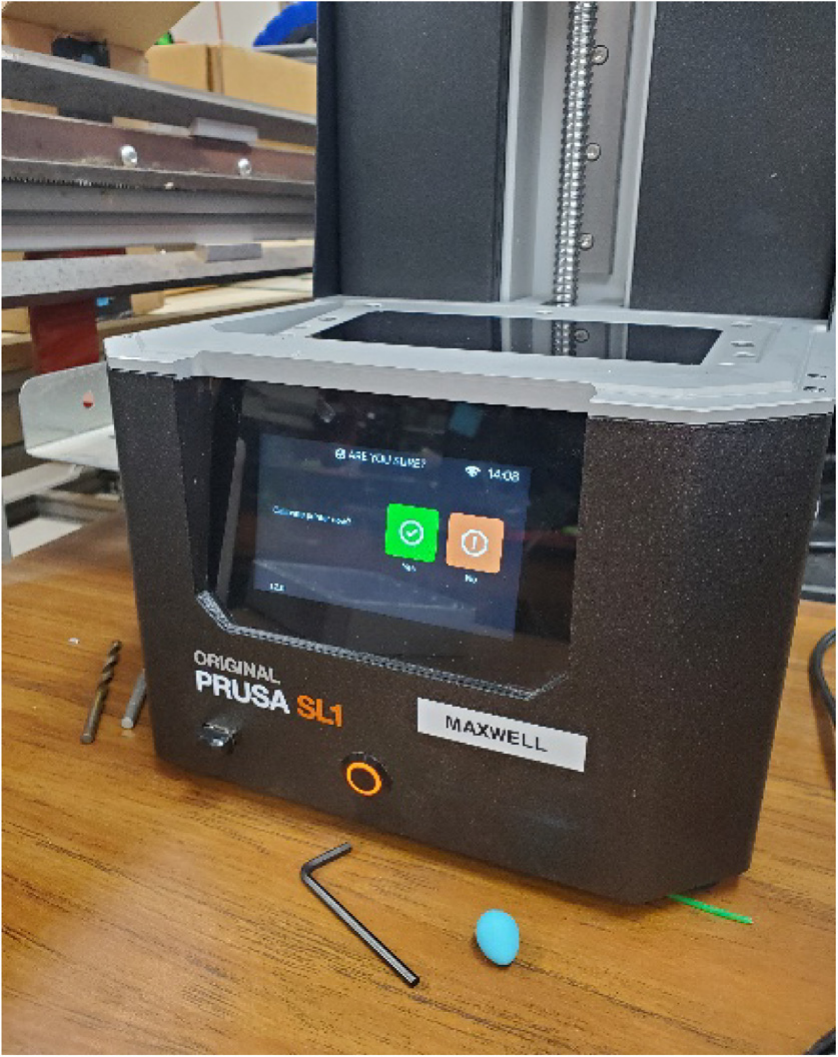
Recalibration Screen.

In order to begin printing on the Prusa SL1 printer, it must be calibrated. Fig. 15 shows the Recalibration Screen.

**Fig. 16:**
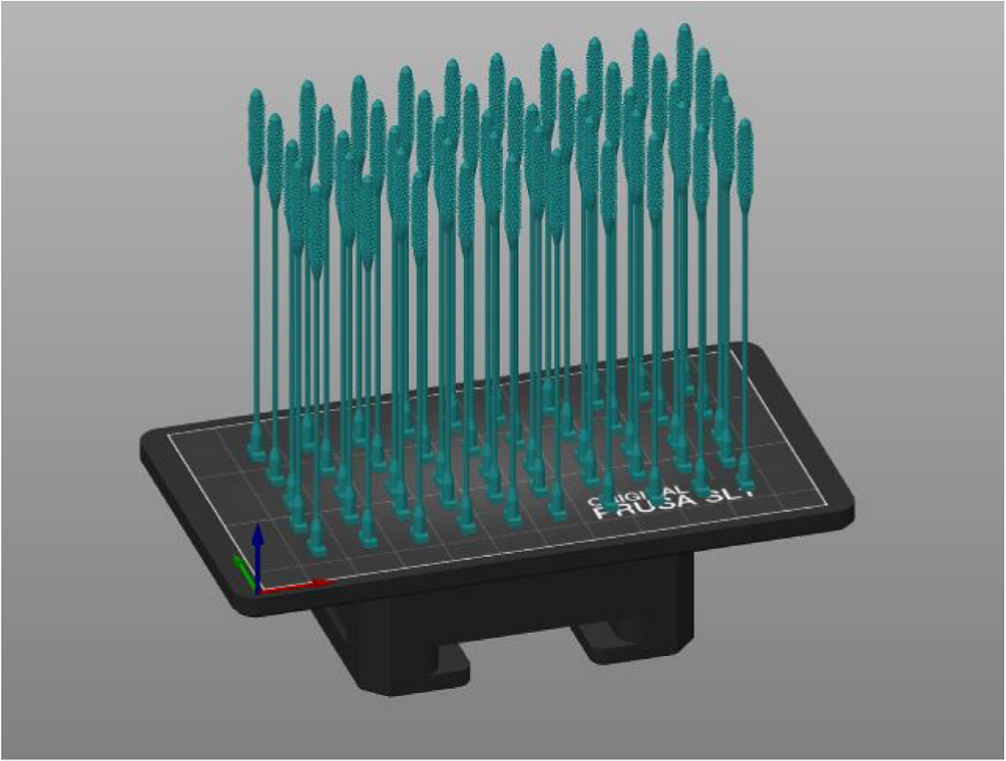
Prusa Print Bed.

The Prusa Slicer (v 2.2.0, Prusa Reseach, Prague, Czech Republic) is open source [64] and is used to import the nasal swab STL file onto the build plate. The design should be multiplied as many times as possible while ensuring a fit on the build plate (Fig. 16). Prints were tested in a 5 by 10 array printing 50 swabs simultaneously.

**Fig. 17:**
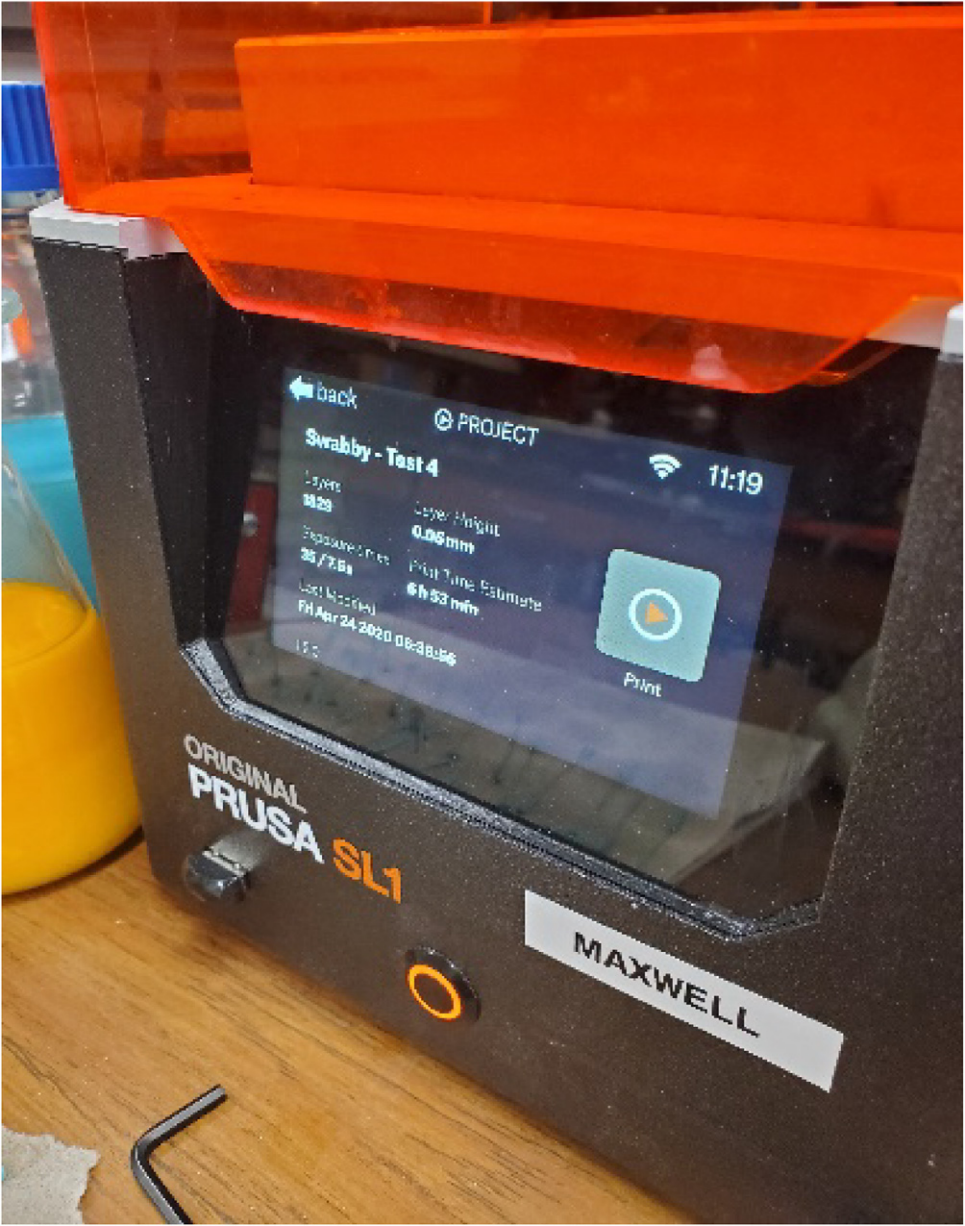
Ready to Print.

When the nasal swab design was sliced, an estimated 13.69 mL of the resin was used. This build plate design was exported onto a flash drive that can be inserted into the Prusa to print. The Prusa will load a screen for how much resin must be in the tank in order to fulfill the requirements for the print (Fig. 17).

**Fig. 18:**
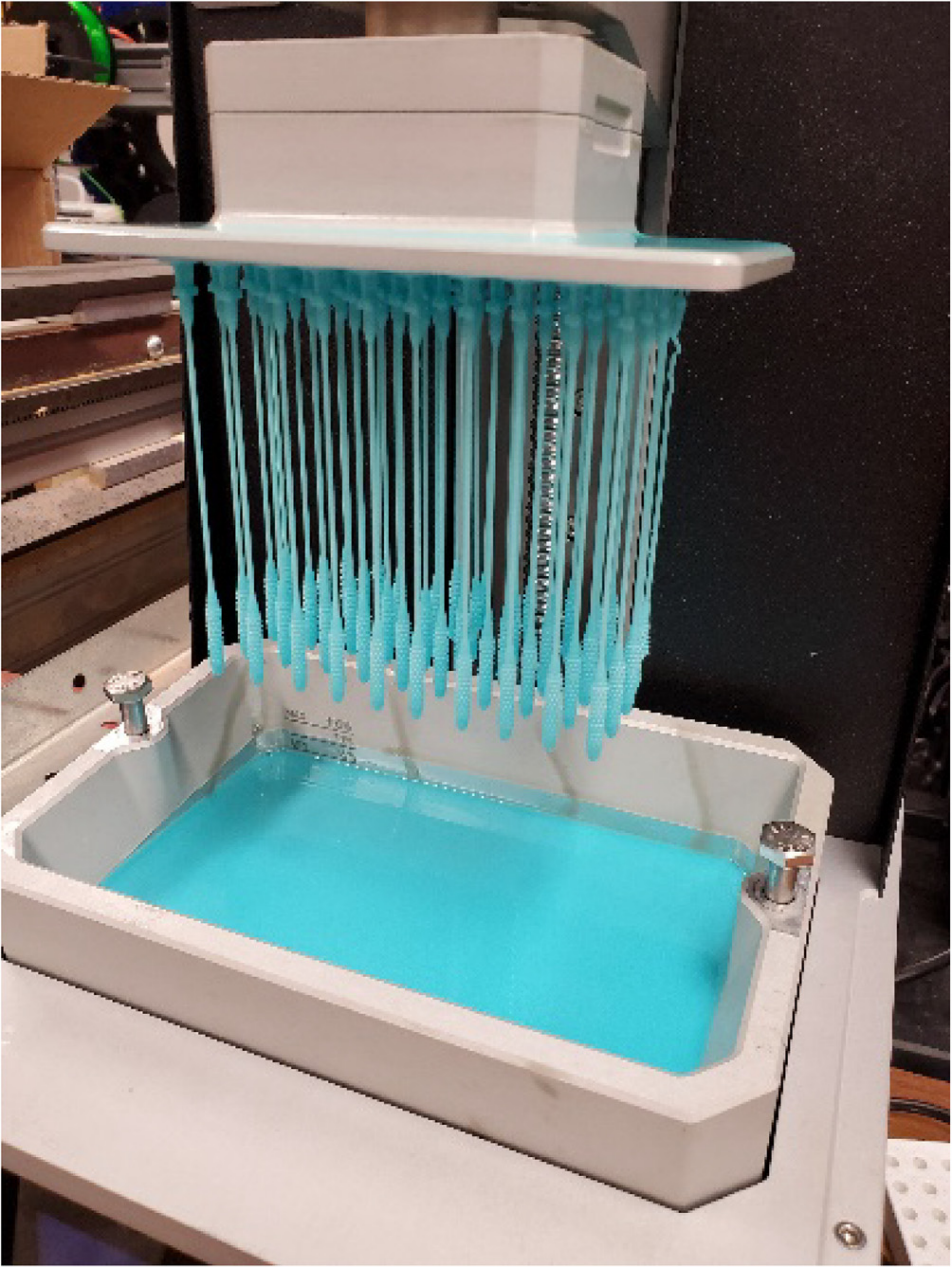
Final Prints.

**Fig. 19:**
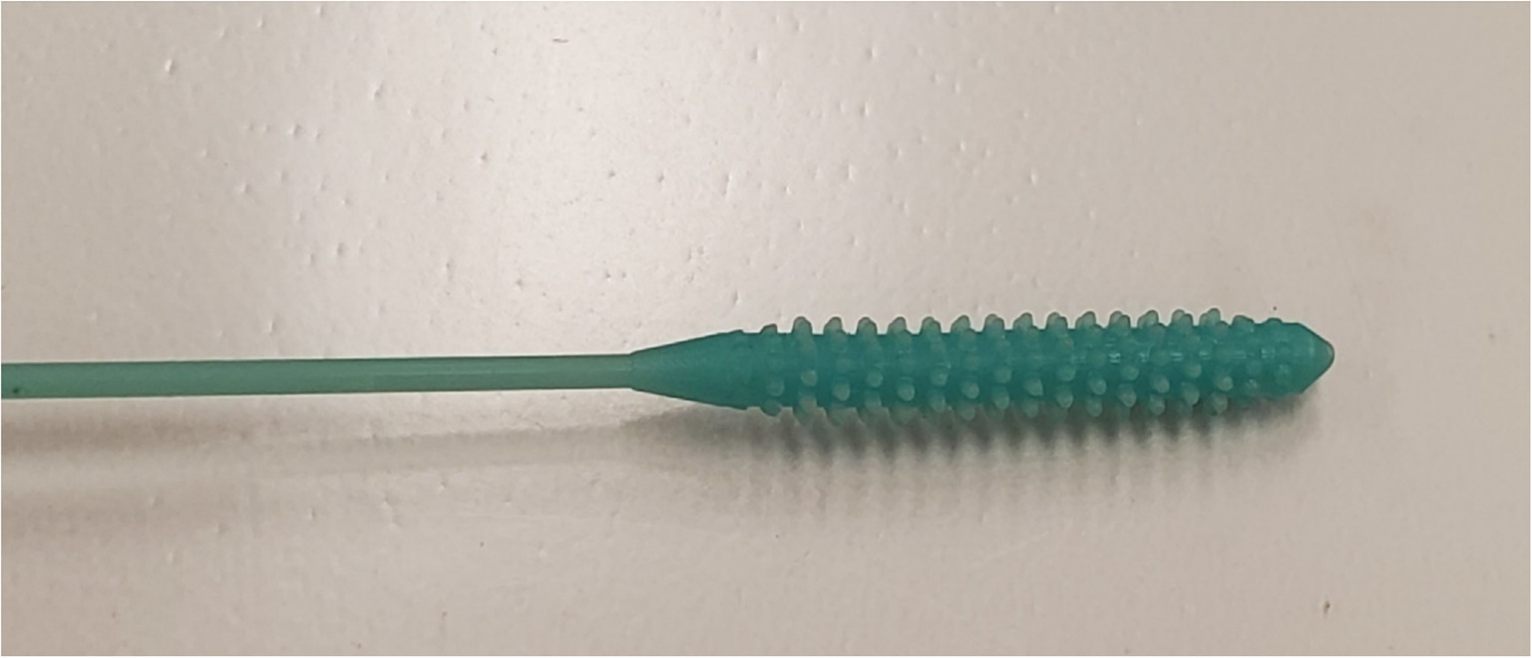
Closeup of swab tip.

The print layer is set to 0.05 mm where the first layer was attached with UV light for 35 s and every consecutive layer was attached for 7 s. The estimated print time for this sequence is 6 h 53 min. This does not include calibration of the printer (which is approximately 5 min) and mixing of the resin once the print begins. Once the print is done, excess resin should be allowed to drip off. After printing, the swabs are removed from the print bed and cleaned with 90% or higher isopropanol and dried by lying flat on a sheet of paper towel (Fig. 18). Each swab was individually rinsed with a squirt bottle while also checking on resolution of the swab head (Fig. 19). This process takes approximately 20 min. The cleaned nasal swabs can either be placed directly in the UV curer or placed on a paper towel to dry. Leftover resin in the basin can be poured into a UV-blocking container, such as a metal water bottle, to be reused once it is filtered. The basin can then be carefully cleaned with paper towel. Warm water and soap can be used to ensure that the film is clean and clear, but metal or isopropanol should not be used to clean the film of the basin. The cleaning process takes approximately 20–30 min.

### 6.3 Swab handle fabrication

**Fig. 20:**
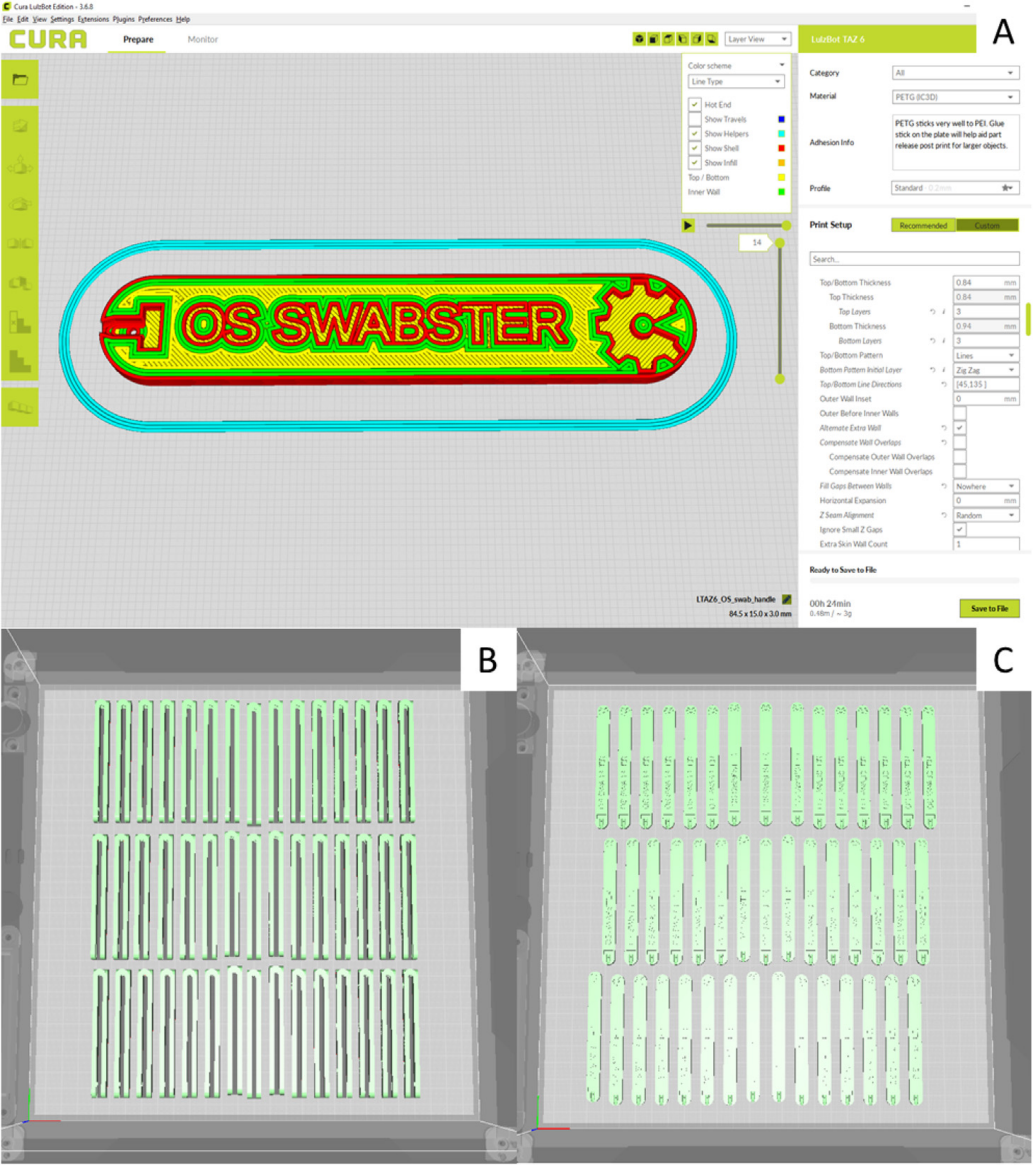
(A) Screen grab of Lulzbot Cura version 3.6.8 of the handle in layer view mode with coloration set to line-type. (B) batches of 45 handles are shown for the tong and (C) press-fit design, respectively.

The swab handle fabrication print parameters should generally follow the normal print settings used as defaults for Lulzbot Cura version 3.6.8 (Fargo Additive Manufacturing Equipment: 3D, Fargo North Dakota, US) [65] for the PLA or PETG materials on the Lulzbot Taz 6. The following settings were changed from the default values and used in the fabrication of the handle for testing purposes: Layer Height: 0.25 mm, Infill Pattern: Zig Zag, Skin Overlap Percentage: 10%, Infill Before Walls: Yes, Retraction of 2.5 mm, Retract before Outer Wall: Yes, Print Speed of 30 mm/s and Regular Fan Speed at layer: 1. The slicer screen is shown in Fig. 20A for an individual press-fit handle and batches of 45 handles are shown in 20B and 20C for the tong and press-fit design respectively. It should be noted that the range of acceptable parameters for the FFF-based 3-D printing for the handle is large and will vary for a given open source 3-D printer, but that the handle is not particularly critical as long as the swab fits well.

**Fig. 21:**
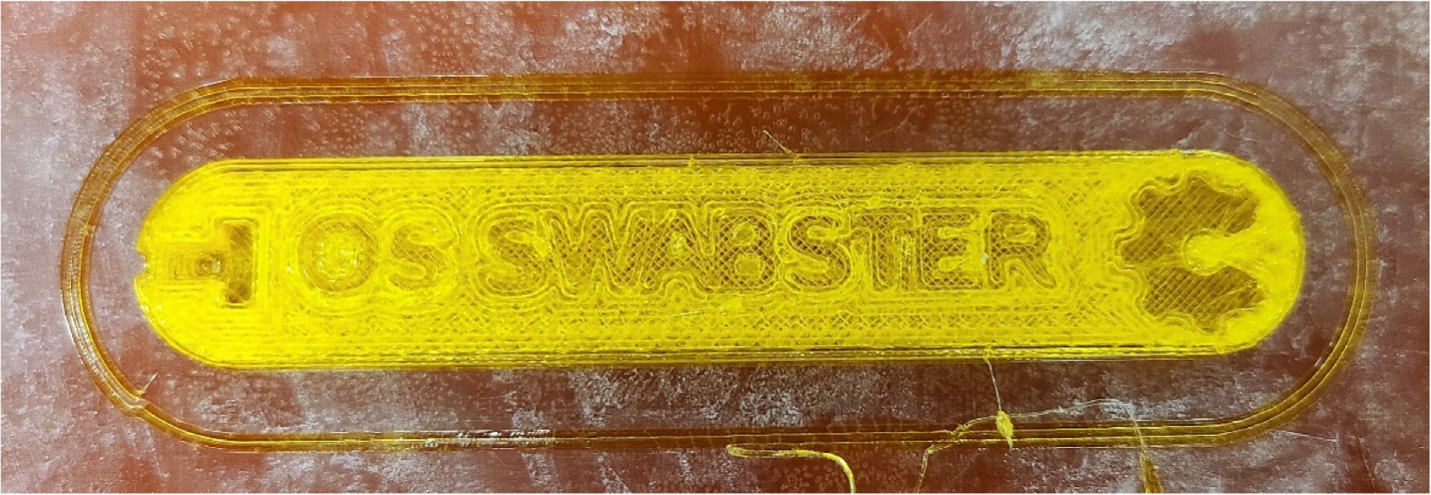
As printed version of handle on a Lulzbot Taz 6 print bed.

**Fig. 22:**
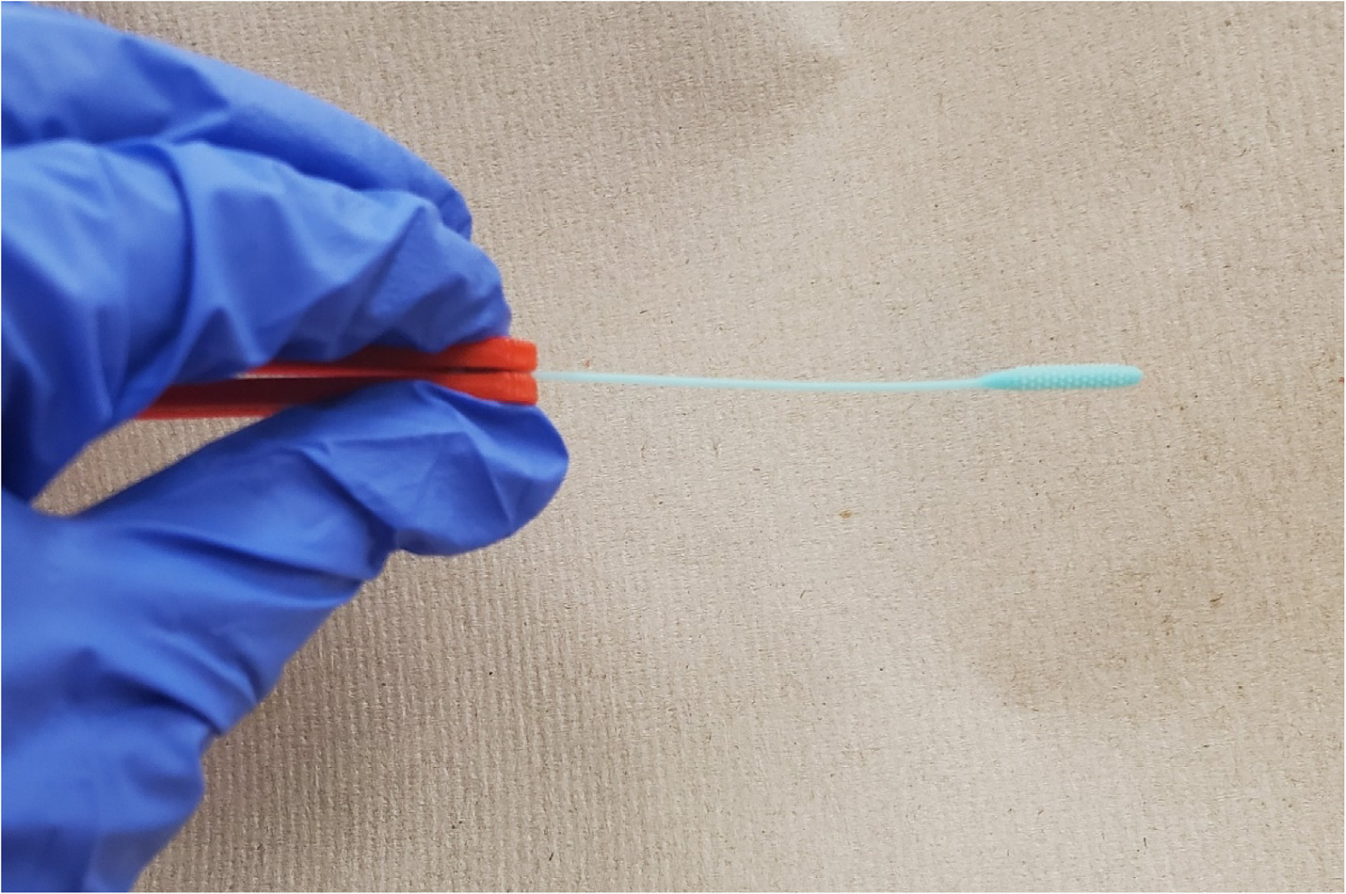
Swab inserted in tong handle.

These parameter changes work for both print materials and both handle versions. A precise outer surface is critical for a clean connection between the press-fit handle and the base section of the nasal swab. The tong handle also benefits from a precise outer surface to both clamp the base of the swab securely and minimize unintended tolerances. Print settings which lower the resolution may lead to a poor fit and inhibit effective use of the swab. In this work, PLA and PETG of multiple colors and brands were used and printed at suggested temperatures by Cura-Lulzbot normal settings. A printed handle post-print is shown in Fig. 21. The swab inserted into a tong handle is shown in Fig. 22. A Lulzbot Taz 6 printer holds 45 press-fit handles, which takes 7 h and 19 min to print. Similarly, on the same print bed 45 tong handles and takes 14 h and 39 min to print. More handles could be fit on the print bed, but these values were chosen to match the SLA printed batches, which would be the limiting factor. Similarly, even more handles could be made in a single print if they were stacked, but based on the print time for 45 this may cause a higher percentage of print failure while adding only modestly to the convenience.

### 6.4 Packaging/sterility

It is well known that the FFF printing process produces sterile objects [66] because of the high temperatures for material extrusion, so if care is taken to chemically sterilize the print bed and use sterile gloves, the handles can be sterile at manufacture. In addition, the chemical compatibility of most commercial 3-D printing filaments has been investigated by Heikkinen et al. [67], which can be used to find an appropriate chemical for any handle material. The swab itself is fabricated using sterile UV light, the post print cleaning is done with isopropyl alcohol that is a disinfection agent and then cured in UV light. Thus, if appropriately handled the swab can remain sterile. Depending on the fabrication facilities and handling the assembled swab and handle may need to be sterilized. The swabs and handles can be sterilized on site and packaged at fabrication or at the hospital. The sterilization should be a non-autoclave process because of the risk of thermal degradation. For example, this can be done with hydrogen peroxide (e.g. the STERRAD process (www.asp.com), which uses hydrogen peroxide vapor and low-temperature gas plasma to sterilize quickly with no toxic residues). The sterilized swabs can be individually packaged in heat sealed plastic pouches, although this was not included in the economic analysis.

## 7 Operation instructions

### 7.1 UV system use

The open source UV curing system being used is made from LEDs and circuit boards and is attached to a 12-volt power supply. The swabs are placed in a 3-D printed holder hanging tip down and placed in the UV curing box and the lid is closed over them (Fig. 14). The box is plugged in and a timer is set for 45 min.

### 7.2 Swab system use

**Fig. 23:**
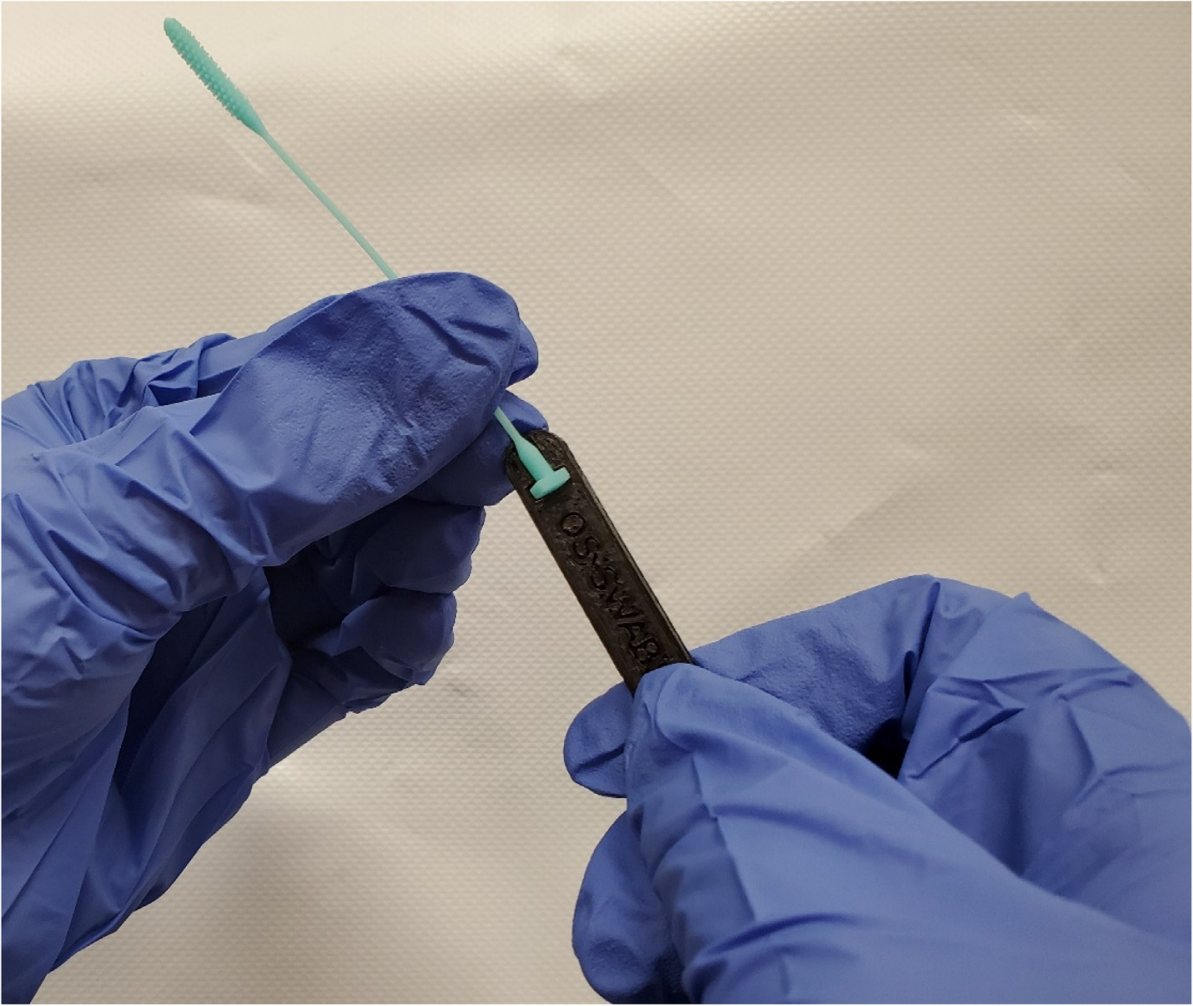
Assembling swab with gloved hands.

Remove swab head from packaging with gloved hands and attach handle as shown in Fig. 23 if it is not already assembled.

**Fig. 24:**
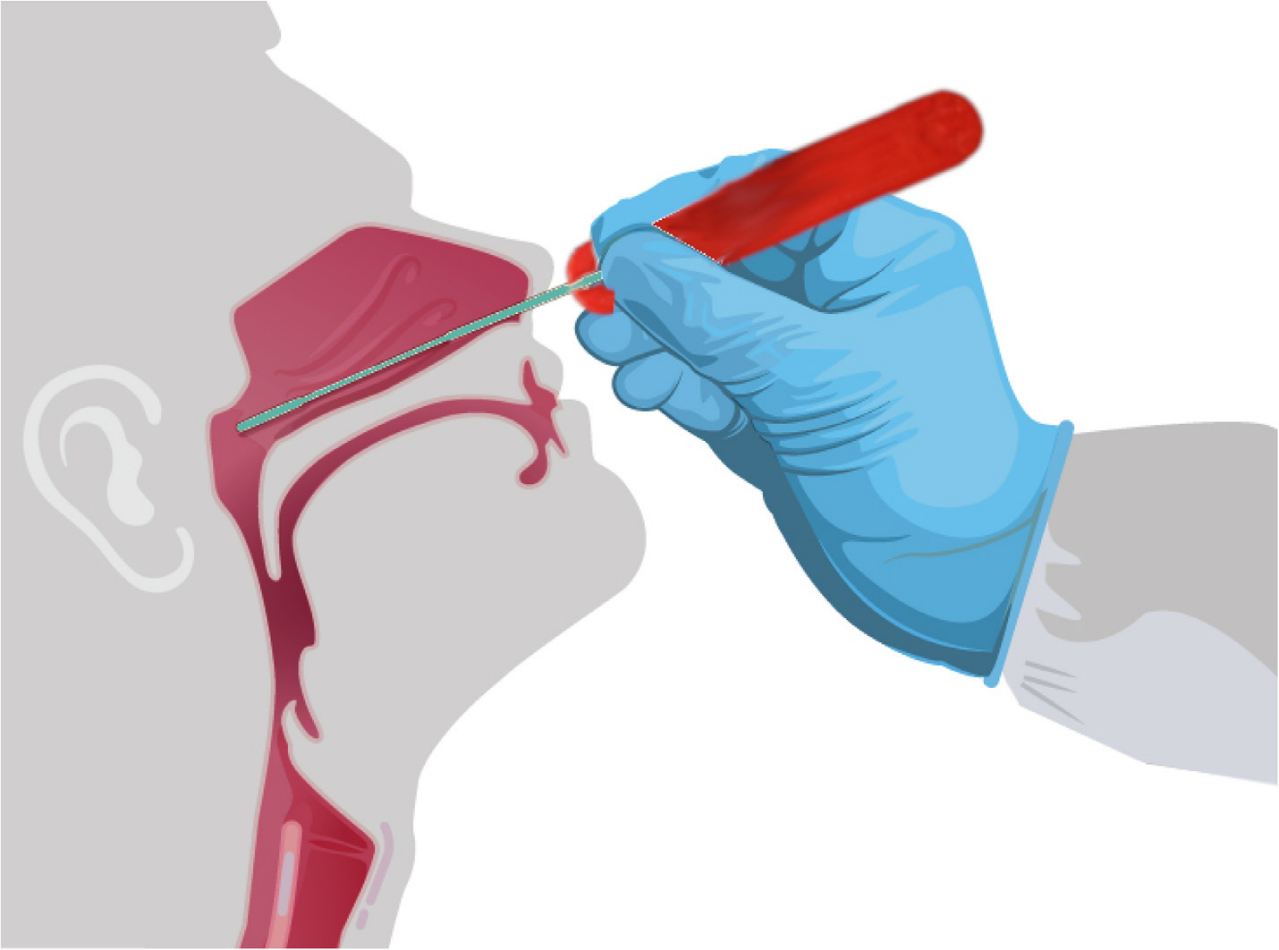
Using the open source nasopharyngeal swab and press-fit handle.

After removing from packaging and if needed, assembling, insert the semi-flexible open source swab through the nares parallel to the palate (not upwards) as shown in Fig. 24 until 1) resistance is encountered indicating contact with the nasopharynx or 2) the distance is equivalent to that from the ear to the nostril of the patient [44]. This area is sensitive so care should be taken. At this location shown in Fig. 24 gently rub the swab and leave it in place for several seconds to absorb secretions. Then, while rotating the swab slowly remove it. After extracting sample following Fig. 24, break off tip of swab and place swab immediately into sterile tubes containing 2–3 mL of viral transport media [44]. See [44] for guidance on storage and shipping samples.

## 8 Validation and characterization

To validate the open source swab, 100 swabs were printed on a Prusa SL1 and underwent the following preclinical testing. The open source nasopharyngeal swabs were mechanically tested for tensile strength, break point and rotation angle at failure and compared to proprietary FormLabs printed nasopharyngeal swabs. The open source nasopharyngeal swabs were also tested for absorption, abrasion and handling testing.

### 8.1 Mechanical and break point testing

Tensile tests were performed on the open source swabs for four different UV curing times: 10 min, 20 min, 30 min and 45 min. Optimum curing times were selected based on the average load during break and standard deviation for 5 specimens. The optimum curing time was then used to cure an array of swabs. 5 specimens were then chosen randomly from the array to test for average load during break and standard deviation. FormLabs samples were also tested for comparison. Tensile testing is meant to mimic pulling the swab out of the nasopharyngeal space.

**Fig. 25:**
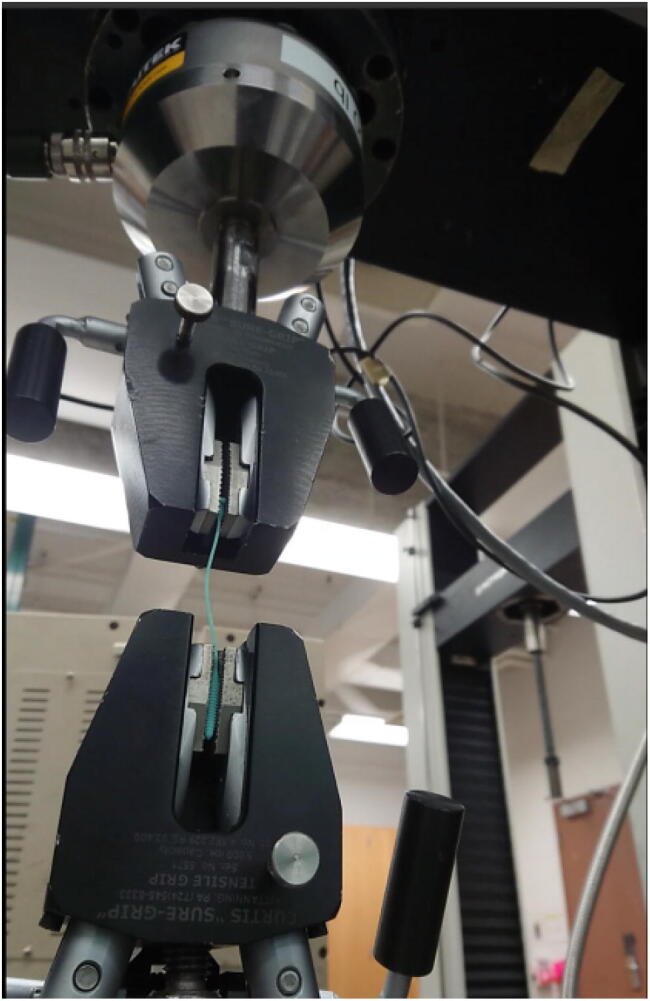
Mechanical testing of swabs with an Instron 4206.

The specimens were tested for tensile strength on Instron 4206 (Nowood, MA, USA) with a 300 lb load cell (Model LCF455, Futek, Irvine, CA, USA) and on Instron 4210 (Nowood, MA, USA) with a 10kN load cell. The results were within error (+/- 1%) on both systems. The setup (Instron 4206) is shown in the Fig. 25.

The Form Labs specimens broke at an average load of 53.67 N with a standard deviation of 3.25 N.

**Fig. 26:**
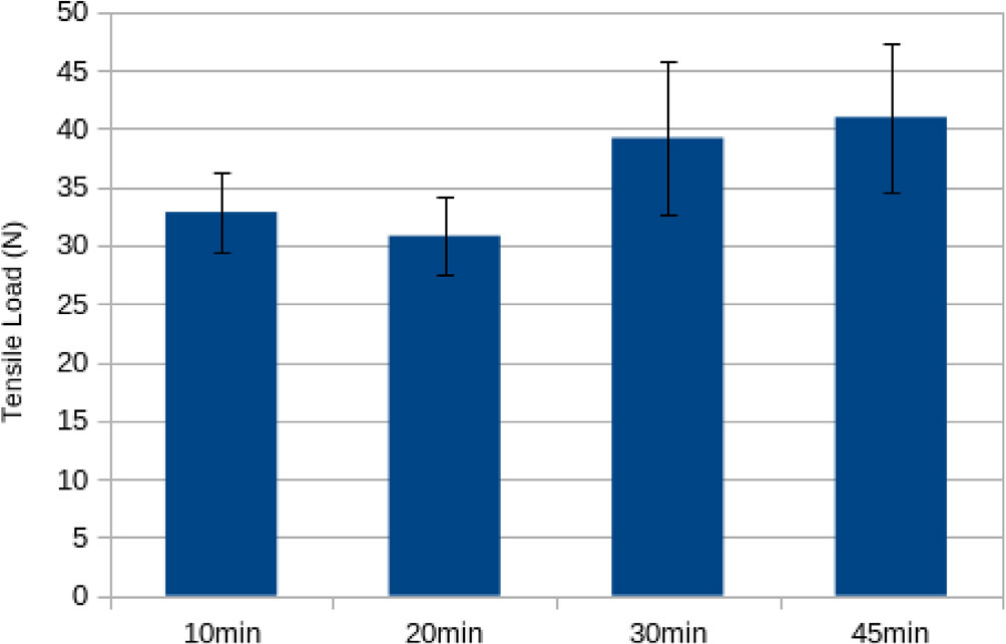
Maximum tensile load at break for open source swabs as a function of UV curing time.

The average load at breaking for the samples at various UV curing times are shown in the Fig. 26. The highest load at break (40.97 N) was at a curing time of 45 min, which was only a slight improvement on the 30 min. cure time and within error of the 30 min cure. This indicates that complete curing has taken place and was thus used throughout the remainder of the testing. It should be noted that all of these forces (40 N is about 4 kg of force) are significantly more than would be expected in a clinical application.

**Fig. 27:**
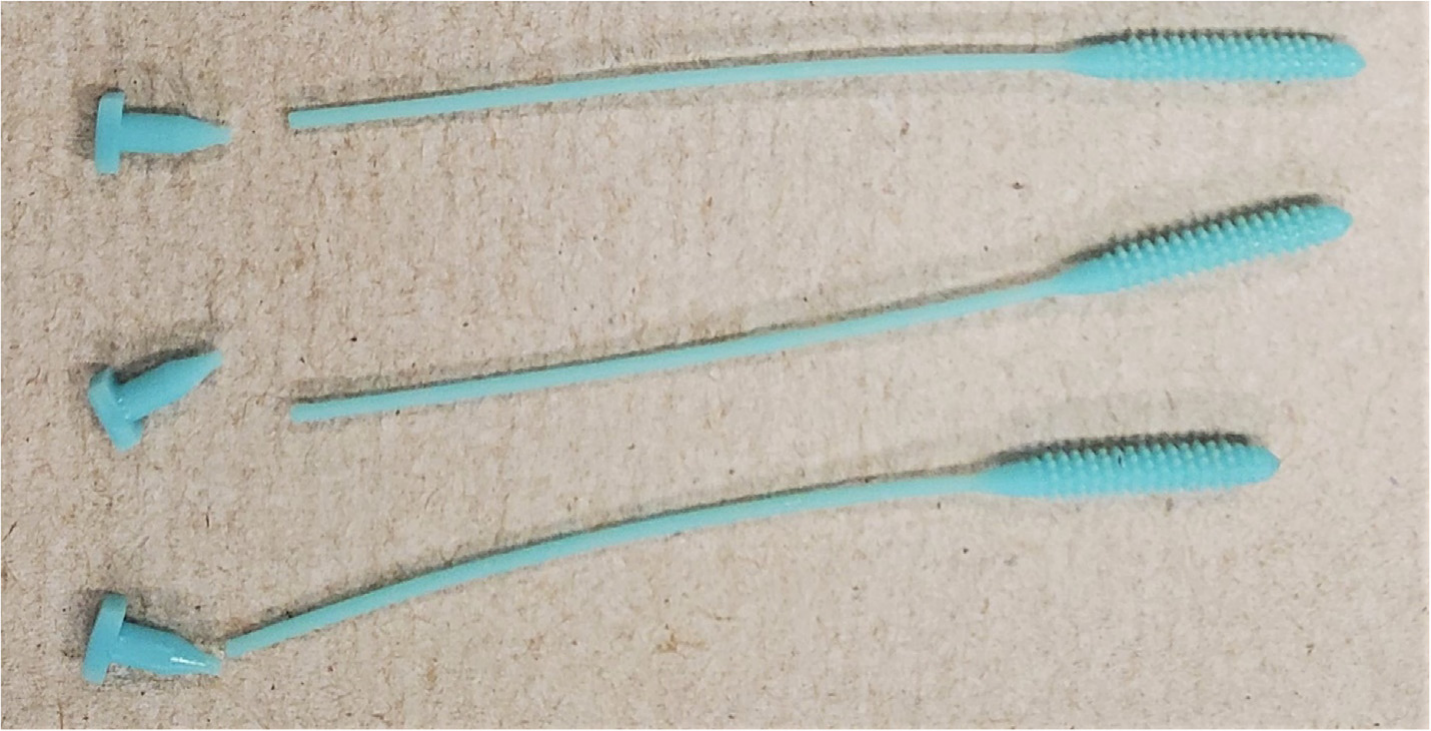
Representative results of break point evaluation.

Although, the open source swabs had a lower tensile strength than the FormLabs swabs, this was purposefully engineered into the design to ensure that the swabs would not break inside of a patient’s nasopharyngeal space. All the Form Labs specimens broke around the middle of the swab. If this were to occur in a patient this would create a new medical problem. All open source swab specimens for all curing times broke at the neck near the handle as designed (hole shown in Fig. 5). Representative examples of this can be seen in Fig. 27.

The position of the swabs in the UV curing box was shown not to be a significant factor in the strength. The specimens chosen from the array of swabs cured at 45 min broke at an average load of 38.02 N with a standard deviation of 5.14 N.

The open source swabs were also tested for rotation until break by placing one end in a vice and rotating. The swabs broke at the break point after about 360 degrees of rotation, which again is far more than would be expected for clinical applications.

### 8.2 Absorption tests

Three different tests were conducted on the open source nasal swabs that were cured for 30 min on each side. The first test was with synthetic nasal mucous (Kryolan, Cotati, CA) [68]. The dry swab was weighed on a calibrated open source digital scale [63]. Then, the swab was placed vertically in synthetic nasal mucous and massed again. Then, the excess nasal mucous is gently rubbed off on paper towel and weighed again in order to determine how much is stored within the tip of the swab when inserted into the nasopharynx. The second test was to capture the nasal mucous by putting it in a petri dish and twisting the swab in a rotational manner. Finally, the third test was done with a honey-water (3:1) mixture that approximated the viscosity of the synthetic nasal mucous so that those developing other swab designs can use more readily available materials (at the time of this writing there was only one synthetic nasal mucous supplier in the U.S.). All experiments were repeated five times.

For the twisting petri dish tests the swabs were found to hold 0.306 g (SD 0.014). For the swabs dipped vertically the swabs held 0.304 g (SD 0.016). These values were found to be comparable with the commercialized FormLabs swabs with an average of 0.297 g. These results indicate that the open source swabs printed on a Prusa SL1 can absorb an adequate amount of material to be used for testing.

The 3:1 honey mixture provided similar results with a mass held between the rough surface of the tip to be 0.314 g (SD 0.023). Although the spread in values was slightly higher with the 3:1 honey mixture these results indicate future swab designers can use honey and water to test absorption in new swabs.

Overall it was found that approximately 0.068 g of material gets caught within the tip of the nasal swab after lightly rolling the swab over a piece of paper towel after dipping in synthetic mucous.

### 8.3 Abrasion tests

Tip abrasion tests were performed by 1) massing the dry weight of the swab on the open source digital scale [63], 2) placing distal end of swab (absorbent end) next to human tissue (inner left arm) and applying a rotation to proximal end (hand side – handle) at hand twirling speed for 10 s, and 3) the swab was removed wash and dried, and 4) massed. The difference in the pre and post abrasion test was recorded. Each experiment was repeated for five trials.

There was no change in mass within error indicating the tips were rugged enough for this application as the arm skin is rougher than that of the inner nasal passage.

### 8.4 Handling testing

**Fig. 28:**
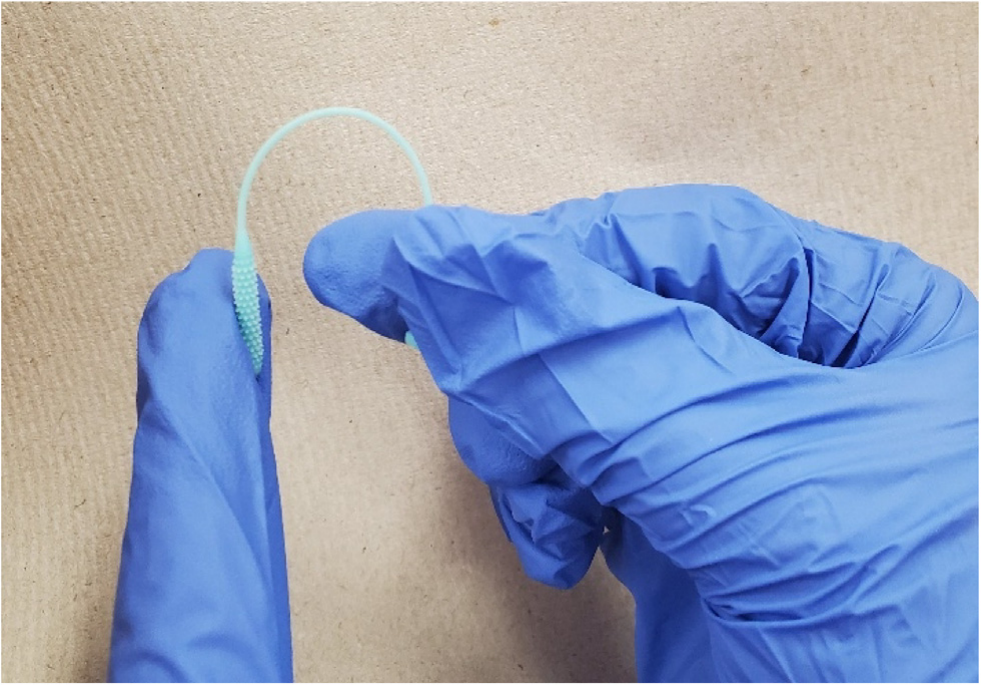
Neck bending test.

**Fig. 29:**
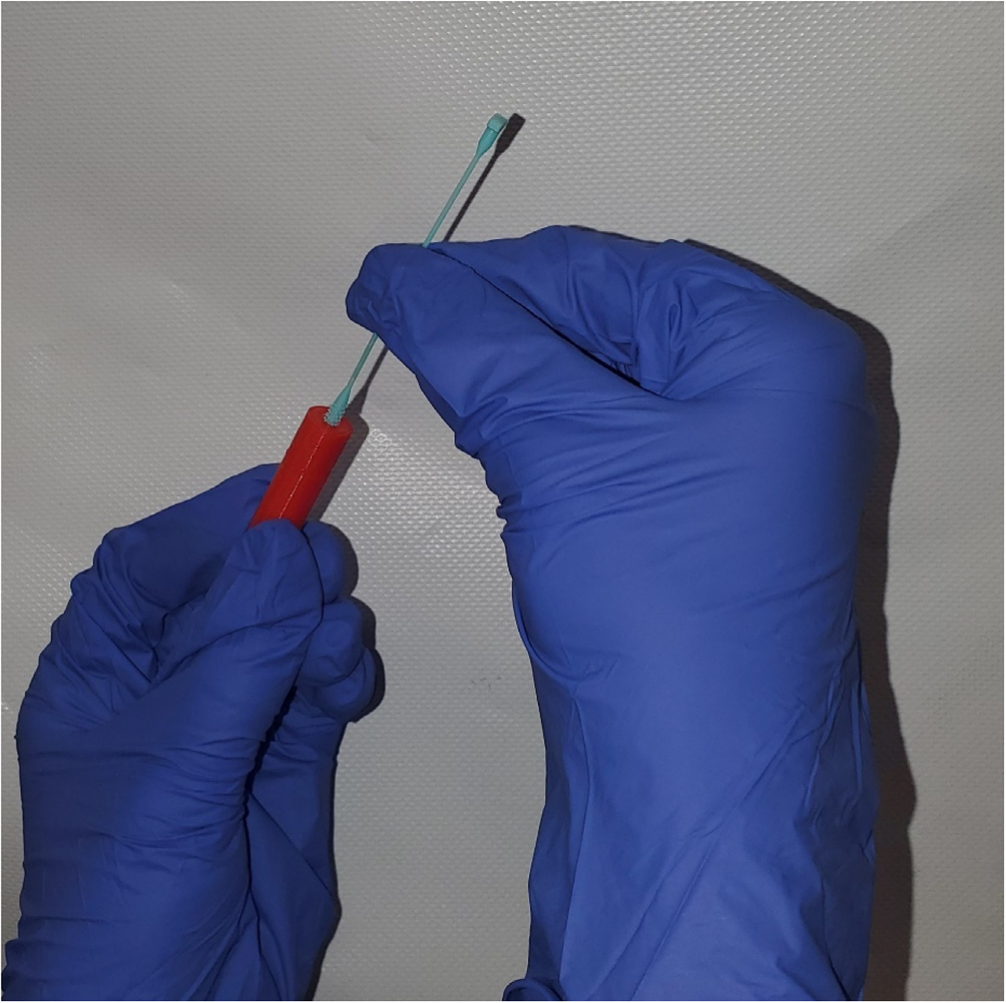
Open source 3-D printed nasal swab tip being tested for robustness to repeated insertions into and removal from a tortuous canal.

Following Callahan et al., [45] the swab heads were tested for head and neck flexibility and robustness to fracture (Fig. 28), robustness to repeat insertion into and removal from a tortuous canal (diameter ~ 3.50 mm (Fig. 29). The neck bending occurred by holding the swab above the break point and bending it to the swab’s nose. Swabs could bend 180 degrees as shown in Fig. 28.

For the robustness to repeated insertions into and removal from a tortuous canal test the tip of the nasal swab was inserted and removed into a 3-D printed channel (https://osf.io/t6yqk/). During both insertion and removal, the swab was rotated slightly by hand as shown in Fig. 29. This process was repeated 20 times. As an end result, there was no visible damage to the swab. Note that the swab needs to be held by the neck past the point where there is an engineered weakness.

Finally, it should be stressed that the swab heads broke at the designed weak point near the insertion point of the handle Fig. 27). The engineered break point can be an issue for the selection of handle type if the swabs are improperly handled. To work the press-fit as designed the swab needs to be pushed into the handle at the T, if not (e.g. pushed from above the break point) it can break at the break at the designed break point point. Open source swab manufacturers may need to adjust the print parameters or design slightly to get a reliable press-fit on their specific FFF/FGF/FPF machine because of varying print tolerances. All of the variables are defined and are adjustable in the OpenSCAD. Minimum angle of fragments and sizes are set at 0.5 and 0.15 respectively with recommendations about changing these variables in the code. Material extrusion users can use standard approaches to fixing either an under or over extrusion that is preventing a good press fit (e.g. recalibrating or adjusting the temperature of their printer). On the other hand, the tongs are more easily fabricated. The tongs are also easy to use, do not require more work on the user’s part, allow the swabber’s fingers to be further from the swabbee’s nose, and make it easy just to drop into viral transport media (VTM). The tongs do have negatives of being costlier and twice as time consuming to produce (because of print time on the FFF/FPF printers) as compared to the press-fit handle. There are some opportunities to reduce this cost by redesigning to be thinner, printing at a lower fill density or shell thickness, but for any given set of print parameters they need a larger volume of material than the press-fit design.

## 9 Safety

Standard safety procedures should be used for FFF-based 3-D printers [69]. For the UV 3-D printers, the casing on the printer must be closed when the print is in progress as exposure to UV light can cause premature skin aging and mutations or eye problems [70]. When using isopropanol to wash the prints, users should wear gloves and eye protective gear [71]. The gloves will protect from possible burning or irritation of the skin. It is flammable, so keep away from flames and high heat.

Nasopharyngeal swabs, which are used to collect specimens from a patient, are Class I devices and are 510(k) exempt [72] from premarket notifications in the U.S. according to 21 CFR § 880.6025 [73]. For the FDA, as of this writing (5–6-2020) 3-D printed swabs are a new technical area that the FDA is working out guidance. Traditional swabs are 510(k) exempt and as such are not generally reviewed by the FDA [74].

Fabricators should check with their local laws, however, as for example the U.S. Food and Drug Administration (FDA) requires medical device manufacturers to register their facility and list their products according to 21 CFR § 807.20 [75]. As of this writing (5–6-2020) this is challenging in the U.S. because the rules differ by state. For example, the Michigan Health Association is running studies with clinical labs across the state [76]. Some public health entities are testing specific safety issues related to some 3-D printed materials. The 3-D printing materials used in this study have not yet been approved of for biological applications and future work is needed to ensure the resin used on the tip is compatible with the human nasopharynx. In addition, although the basic makeup of the resin for the tip and hard plastics for the handle used here were known, the pigments and any other additives was not disclosed. This problem has been discussed before [77] in the context of expanding John F. Kennedy’s Consumer Bill of Rights to include material ingredients [78]. This application of distributed manufacturing using 3-D printing for critical medical devices during a pandemic [79] further underscores the necessity to have all materials be openly available and disclosed for all products, but particularly for those used in manufacturing.

In addition, as the results here clearly showed that after post-printing uv curing, the mechanical properties of the swabs improve. This means that there are mobile chemicals and monomers within the printed structure that have the potential to irritate human tissue as the uncured material can be harmful as a respiratory irritant [80]. Future work is needed to verify the presence of free monomers or chemicals to indicate for how long they can be extracted out while immersed in IPA. Similarly, this design with a FFF/FPF/FGF printed handle was made without an intension of reuse. This is because the ability to sterilize material extrusion parts is limited because of their low melting temperatures of often used feedstocks (e.g. PLA, ABS, PETG) restricts the use of thermal sterilization. Sterilization is possible with autoclave, ethylene oxide, hydrogen peroxide and gamma radiation; although the former causes major deformation and the others present challenges [81]. 3-D printed materials can be compatible with H_2_O_2_ vapor sterilization, but again there are substantial challenges [82]. Another approach is to use an open source high-temperature printer to fabricate the handles in polyetherketoneketone (PEKK) or polyetherimide (PEI, ULTEM), which would allow for thermal sterilization [83]. Although the open source printer can be manufactured for $1,000 in parts it is new and other high-temperature material extrusion printers are high cost and thus also not as well-dispersed as standard desktop FFF-based machines.

These biological compatibility experiments are needed to show the safety of the 3-D printing materials before this approach can be used clinically. As NP swabs fall under a specific device class, the FDA has a running list online of specific manufacturers and types of materials acceptable for use in COVID responses. This presents a challenge to distributed manufacturers. Currently, Michigan MDHHS, BOL have been using the list for what they can use. It is up to each and every lab director to make their own decisions and turn in appropriate paperwork documentation to FDA regarding what they choose to use based on how it fits with their assay EUA documents (some platforms have more restrictions than others) [76]. This obviously provides a bureaucratic barrier to acting quickly, which in part explains the limited supply of swabs in Michigan. This work should help expedite distributed manufacture of nasal swab use for future pandemics or supply disruptions.

## 10 Discussion

A nasal swab goes into the nose about 4–6 cm from the nasal opening to the back of the nasopharynx. A good rule of thumb is the distance from the opening of the nose to the opening of the ear (measured on the outside). Commercial swabs have a relatively firm 10.5 cm long handle that is approximate 2.5 mm diameter. This attaches to a flexible 3.5 cm in length neck 1 mm diameter, where the break point is. This in turn is attached to the fibrillar head, which is 2.0 cm in length, with an approximate 3 mm diameter. The variance in the SLA prints is not a factor in this application (e.g. the diameter of the head).The proprietary 3-D printed swab tested (the Northwell Swab STL) with the base handle cut off (97%) is approximately 97.15 mm. The total length of the swab would therefore be about 100 mm (100.0645 mm). The final dimensions of the open source swab are: length of 85.20 mm, diameter at the base is 2.45 mm, length of the neck is 54.60 mm with a 1.05 mm diameter and length of the head is 22.30 mm with a diameter of 3.30 mm. Both the tong and press-fit handles are 80 mm in length, creating a total for the new swab and handle to be approximately 156.57 mm since the base of the swab is held in the handle. The open source design provided here is able to meet these specifications with a broad flexibility of using the parametric design to meet different types of swab needs.

The results of the mechanical validation tests showed that the swabs could withstand greater forces than would be expected in normal clinical use. The swabs were also able to absorb a significant amount of material and passed the abrasion and handling tests. The open source swab therefore appears to be a promising candidate for clinical trials.

The single swab stl can be copied until an entire print bed is filled in the PrusaSlicr Program. In this study, the Prusa print bed was organized in a 5x10 swab fashion (50 swabs per print), to allow each swab build space towards the fibular tip. Future work could evaluate printing the swabs at a higher density and filling the entire build platform. At the same time, the quality of a particular print is important for this application so a low-cost rapid method of quality verification can be developed as future work as well.

The bottle neck SLA 3-D print time for the open source swab is 6 h 45 min for 50 swabs. For the entire process from calibration to cleaning of the swabs, is approximately 7.5 h. So, the throughput of the SLA portion of the swab represents about 9 min/swab/machine. The ability to use lower cost UV curable resin from Prusa or other open materials suppliers also represents potential cost savings compared to proprietary SLA systems although future work on the yields of those systems would need to be completed for a full cost comparison. As there is significant time and cost savings using a two-part design the economics for other SLA printers would improve for printing swabs if a similar design to the one shown here is used.

Another distinct advantage to this two-part system is the ability to brand or customize the handle. This feature could be used to cut down on waste. Normally swabs are discarded as medical waste and incinerated. As only the head of the open source swab goes to the lab for testing the handles could be either sterilized and reused as handles or sterilized and reused for other applications. An example might be to make a handle with Lego compatible geometries so that sterilized swab handles could be cleaned and then used in children’s waiting rooms at the hospital as building blocks.

This design is fully licensed as open source (as defined by the Open Source Hardware Association (OSHWA) [29]), which comes with distinct benefits for scaled distributed manufacturing as well as enabling creativity and productivity in the case of future pandemics or needs. In general, the designs in clinical use are proprietary and provided with a license waiver on a high-cost proprietary tool. This provides the opportunity for some emergency distributed manufacturing, but it is limited, mostly by access to the high-cost tooling. There are freely available (accessible) untested designs that are creative commons licensed with a non-commercial license (for example see untested accessible designs [84], [85], [86], [87]). These designs are thus not strictly open source by the OSHWA definition as they restrict manufacturers from selling them, which could limit deployment as for example distributed manufacturers would not be able to recoup the cost of materials or their personnel time.

Lastly, and most importantly the open source swabs need to be verified clinically before widespread use. The State of Michigan has a Validation Plan [88], which includes a 3-D Printed Swab Bridging Study. To bridge the performance of novel 3-D printed swabs, the FDA recommends [89] testing 3-fold serial dilutions of SARS-CoV-2 viral material in pooled respiratory sample matrix in triplicate in parallel between the new and existing component, where the smallest serial dilution should be no greater than 1-2X the demonstrated limit of detection of the assay. According to the FDA, acceptance criteria is 95% concordance between the two components. In Michigan each laboratory will determine the parameters to evaluate to assess agreement based on the technical performance of their assays. These tests will need to be completed for the open source nasopharyngeal swabs for the specific UV-curable materials used. 3-D printer operators can work with clinicians to assess suitability of prospective products for use in local patient care settings.

## Declaration of Competing Interest

The authors declare that they have no known competing financial interests or personal relationships that could have appeared to influence the work reported in this paper.

## References

[1] World Health Organization, 2020. Critical preparedness, readiness and response actions for COVID-19: interim guidance, 7 March 2020 (No. WHO/COVID-19/Community_Actions/2020.1). World Health Organization.

[2] Silv, M., COVID-19: too little, too late?. The Lancet. doi: 10.1016/S0140-6736(20)30522-5

[3] Fisher D., Heymann D. (2020). Q&A: The novel coronavirus outbreak causing COVID-19. BMC Med..

[4] L. Ramsey, Hospitals could be overwhelmed with patients and run out of beds and ventilators as the coronavirus pushes the US healthcare system to its limits. Mar 11, 2020, 8:53 AM Business Insider. https://www.businessinsider.com/coronavirus-intensive-care-unit-shortages-of-ventilators-staff-space-2020-3?op=1.

[5] Rubinson L., Vaughn F., Nelson S., Giordano S., Kallstrom T., Buckley T., Burney T., Hupert N., Mutter R., Handrigan M., Yeskey K. (2010). Mechanical ventilators in US acute care hospitals. Disaster Med. Public Health Preparedness.

[6] J. Miller, Germany, Italy rush to buy life-saving ventilators as manufacturers warn of shortages. Reuters. 2020. https://www.reuters.com/article/us-health-coronavirus-draegerwerk-ventil-idUSKBN210362

[7] P. Neighmond, As The Pandemic Spreads, Will There Be Enough Ventilators?,” 2020. NPR. https://www.npr.org/sections/health-shots/2020/03/14/815675678/as-the-pandemic-spreads-will-there-be-enough-ventilators.

[8] Cook T.M. (2020). Personal protective equipment during the COVID-19 pandemic–a narrative review. Anaesthesia.

[9] Livingston E., Desai A., Berkwits M. (2020). Sourcing personal protective equipment during the COVID-19 pandemic. JAMA.

[10] Ranney M.L., Griffeth V., Jha A.K. (2020). Critical supply shortages — the need for ventilators and personal protective equipment during the Covid-19 pandemic. N. Engl. J. Med..

[11] WHO. Shortage of personal protective equipment endangering health workers worldwide [WWW Document], 2020. URL https://www.who.int/news-room/detail/03-03-2020-shortage-of-personal-protective-equipment-endangering-health-workers-worldwide (accessed 4.22.20).

[12] Jacobs A., Richtel M., Baker M. (2020).

[13] K. Siegler, 2020. Many Who Need Testing For COVID-19 Fail To Get Access [WWW Document], NPR.org. URL https://www.npr.org/2020/04/03/826044608/many-who-need-testing-for-covid-19-fail-to-get-access (accessed 4.22.20).

[14] Collins, K., 2020. Coronavirus Testing Needs to Triple Before the U.S. Can Reopen, Experts Say. The New York Times.

[15] D. Lim, Latest coronavirus testing glitch: Not enough cotton swabs [WWW Document], POLITICO. URL https://www.politico.com/news/2020/03/16/coronavirus-testing-glitch-cotton-swabs-132692 (accessed 4.22.20), 2020.

[16] E. Blake, M. Hicken, A. Fantz, Coroners worry Covid-19 test shortages could lead to uncounted deaths [WWW Document]. CNN. URL https://www.cnn.com/2020/04/06/health/coronavirus-coroners-uncounted-deaths-invs/index.html (accessed 4.22.20), 2020.

[17] Memorandum on Order Under the Defense Production Act Regarding 3M Company [WWW Document], 2020. The White House. URL https://www.whitehouse.gov/presidential-actions/memorandum-order-defense-production-act-regarding-3m-company/ (accessed 4.22.20).

[18] A.M. Ollstein, 2020. Trump invokes DPA for testing swabs, weeks after reported shortages [WWW Document], POLITICO. URL https://www.politico.com/news/2020/04/19/trump-dpa-testing-swabs-reported-shortages-195721 (accessed 4.22.20).

[19] Wittbrodt B.T., Glover A.G., Laureto J., Anzalone G.C., Oppliger D., Irwin J.L., Pearce J.M. (2013). Life-cycle economic analysis of distributed manufacturing with open-source 3-D printers. Mechatronics.

[20] Srai J.S., Kumar M., Graham G., Phillips W., Tooze J., Ford S., Beecher P., Raj B., Gregory M., Tiwari M.K., Ravi B. (2016). Distributed manufacturing: scope, challenges and opportunities. Int. J. Prod. Res..

[21] Laplume A., Anzalone G.C., Pearce J.M. (2016). Open-source, self-replicating 3-D printer factory for small-business manufacturing. Int. J. Adv. Manufacturing Technol..

[22] Stacey M. (2014). The FAB LAB network: A global platform for digital invention, education and entrepreneurship. Innovations: Technology, Governance, Globalization.

[23] Byard D.J., Woern A.L., Oakley R.B., Fiedler M.J., Snabes S.L., Pearce J.M. (2019). Green fab lab applications of large-area waste polymer-based additive manufacturing. Addit. Manuf..

[24] DeVor R.E., Kapoor S.G., Cao J., Ehmann K.F. (2012). Transforming the landscape of manufacturing: distributed manufacturing based on desktop manufacturing (DM) 2. J. Manuf. Sci. Eng..

[25] Gwamuri J., Wittbrodt B.T., Anzalone N.C., Pearce J.M. (2014). Reversing the trend of large scale and centralization in manufacturing: The case of distributed manufacturing of customizable 3-D-printable self-adjustable glasses. Challenges in Sustainability.

[26] Woern A.L., Pearce J.M. (2017). Distributed manufacturing of flexible products: Technical feasibility and economic viability. Technologies.

[27] Petersen E.E., Pearce J. (2017). Emergence of home manufacturing in the developed world: Return on investment for open-source 3-D printers. Technologies.

[28] Gibb A., Abadie S. (2014).

[29] OSHWA. 2020. Definition (English) – Open Source Hardware Association [WWW Document], n.d. URL https://www.oshwa.org/definition/ (accessed 4.22.20).

[30] Oberloier S., Pearce J.M. (2018). General design procedure for free and open-source hardware for scientific equipment. Designs.

[31] Ventola C.L. (2014). Medical applications for 3D printing: current and projected uses. Pharmacy and Therapeutics.

[32] Niezen G., Eslambolchilar P., Thimbleby H. (2016). Open-source hardware for medical devices. BMJ Innovations.

[33] Michaels R.E., Pearce J.M. (2017). 3-D printing open-source click-MUAC bands for identification of malnutrition. Public Health Nutr..

[34] Pearce J.M. (2017). Maximizing returns for public funding of medical research with opensource hardware. Health Policy Technol..

[35] Tatham P., Loy J., Peretti U. (2015). Three dimensional printing–a key tool for the humanitarian logistician?. J. Humanitarian Logistics Supply Chain Manage..

[36] S. Saripalle, H. Maker, A. Bush, N. Lundman, 2016, October. 3D printing for disaster preparedness: Making life-saving supplies on-site, on-demand, on-time. In 2016 IEEE Global Humanitarian Technology Conference (GHTC) (pp. 205-208). IEEE.

[37] James E., James L. (2016). 3D printing humanitarian supplies in the field. Humanit. Exch.

[38] Savonen B.L., Mahan T.J., Curtis M.W., Schreier J.W., Gershenson J.K., Pearce J.M. (2018). Development of a resilient 3-D printer for humanitarian crisis response. Technologies.

[39] D. Kats, L. Spicher, B. Savonen, J. Gershenson, Paper 3D Printing to Supplement Rural Healthcare Supplies—What Do Healthcare Facilities Want?, in: 2018 IEEE Global Humanitarian Technology Conference (GHTC) (pp. 1-8). IEEE, 2018, October.

[40] R.M. Madrigal, C. Alexis, A New Statistic Reveals Why America’s COVID-19 Numbers Are Flat [WWW Document]. The Atlantic. URL https://www.theatlantic.com/technology/archive/2020/04/us-coronavirus-outbreak-out-control-test-positivity-rate/610132/ (accessed 4.22.20), 2020.

[41] Salathé M., Althaus C.L., Neher R., Stringhini S., Hodcroft E., Fellay J., Zwahlen M., Senti G., Battegay M., Wilder-Smith A., Eckerle I. (2020). COVID-19 epidemic in Switzerland: on the importance of testing, contact tracing and isolation. Swiss Medical Weekly.

[42] Wu Z., McGoogan J.M. (2020). Characteristics of and important lessons from the coronavirus disease 2019 (COVID-19) outbreak in China: summary of a report of 72 314 cases from the Chinese Center for Disease Control and Prevention. JAMA.

[43] U. Irfan, The case for ending the Covid-19 pandemic with mass testing [WWW Document]. Vox. URL https://www.vox.com/2020/4/13/21215133/coronavirus-testing-covid-19-tests-screening (accessed 4.22.20), 2020.

[44] CDC, 2020. Interim Guidelines for Collecting, Handling, and Testing Clinical Specimens from Persons for Coronavirus Disease 2019 (COVID-19). Coronavirus Disease 2019 (COVID-19) [WWW Document]. Centers for Disease Control and Prevention. URL https://www.cdc.gov/coronavirus/2019-ncov/lab/guidelines-clinical-specimens.html (accessed 4.22.20).

[45] C.J. Callahan, R. Lee, K. Zulauf, L. Tamburello, K.P. Smith, J. Previtera, A. Cheng, A. Green, A.A. Azim, A. Yano, N. Doraiswami, J. Kirby, R. Arnaout, Open Development and Clinical Validation of Multiple 3D-Printed Sample-Collection Swabs: Rapid Resolution of a Critical COVID-19 Testing Bottleneck. medRxiv 2020.04.14.20065094, 2020. doi: 10.1101/2020.04.14.20065094. 10.1128/JCM.00876-20 PMC7383530 32393482

[46] Home | COVID Swabs [Internet]. [cited 2020 May 15]. Available from: https://printedswabs.org/

[47] Covidswab/BIDMC at master · rarnaout/Covidswab · GitHub [Internet]. [cited 2020 May 15]. Available from: https://github.com/rarnaout/Covidswab/tree/master/BIDMC.

[48] USF Nasal Swab [Internet]. [cited 2020 May 15]. Available from: https://usf.app.box.com/s/wxmlj0r66vp8bzei6o7sur1kq1jr8o1i.

[49] rarnaout. rarnaout/Covidswab [Internet]. 2020 [cited 2020 May 15]. Available from: https://github.com/rarnaout/Covidswab.

[50] Formlabs. 2020. 3D Printed COVID-19 Test Swabs [WWW Document], Formlabs. URL https://formlabs.com/covid-19-response/covid-test-swabs/ (accessed 4.22.20).

[51] Form 3 and Form 3L [WWW Document], n.d. . Formlabs. URL https://formlabs.com/store/form-3/ (accessed 4.22.20).

[52] USF Health. Northwell Health. [WWW Document], n.d. https://usf.app.box.com/s/wxmlj0r66vp8bzei6o7sur1kq1jr8o1i/file/649113591064 (accessed 4.22.20).

[53] OpenSCAD [WWW Document], 2020. URL http://openscad.org (accessed 4.22.20).

[54] Original Prusa SL1 – Prusa3D – 3D Printers from Josef Průša [Internet]. [cited 2020 Apr 22]. Available from: https://www.prusa3d.com/original-prusa-sl1/

[55] Tanikella N.G., Wittbrodt B., Pearce J.M. (2017). Tensile strength of commercial polymer materials for fused filament fabrication 3D printing. Addit. Manuf..

[56] Woern A.L., Byard D.J., Oakley R.B., Fiedler M.J., Snabes S.L., Pearce J.M. (2018). Fused particle fabrication 3-D printing: recycled materials’ optimization and mechanical properties. Materials.

[57] Jones R., Haufe P., Sells E., Iravani P., Olliver V., Palmer C., Bowyer A. (2011). RepRap–the replicating rapid prototyper. Robotica.

[58] E. Sells, S. Bailard, Z. Smith, A. Bowyer, V. Olliver, RepRap: the replicating rapid prototyper: maximizing customizability by breeding the means of production, in: Handbook of Research in Mass Customization and Personalization: (In 2 Volumes) (pp. 568-580), 2010.

[59] A. Bowyer, 3D printing and humanity's first imperfect replicator. 3D printing and additive manufacturing, 1(1), pp.4-5, 2014.

[60] LulzBot Taz 6 | LulzBot [WWW Document], n.d. URL https://www.lulzbot.com/store/printers/lulzbot-taz-6 (accessed 4.22.20).

[61] Universal transport media collection Kit with swab - Buy Product on vegas Available online: http://www.vegasbiotech.com/Universal-Transport-Medium-with-flocked-swab-pd42752296.html (accessed on May 14, 2020).

[62] Amazon.com: Chanzon 100 pcs 5mm Purple UV LED Diode Lights (Violet Clear Round Transparent DC 3V 20mA) Bright Lighting Bulb Lamps Electronics Components Indicator Light Emitting Diodes: Home Improvement Available online: https://www.amazon.com/Ultraviolet-Lighting-Electronics-Components-Emitting/dp/B01AUI4VTM (accessed on May 14, 2020).

[63] B.R. Hubbard, J.M. Pearce, Open Source Digitally Replicable Lab-Grade Scales. Preprints 2020, 2020040472 (doi: 10.20944/preprints202004.0472.v1).

[64] PrusaSlicer – Prusa3d.com – 3D printers by Josef Prusa [Internet]. Prusa3D – 3D Printers from Josef Průša. [cited 2020 May 15]. Available from: https://www.prusa3d.com/prusaslicer/; full source doe available: https://github.com/prusa3d/PrusaSlicer/releases.

[65] Cura LulzBot Edition [Internet]. LulzBot. 2015 [cited 2020 May 15]. Available from: https://www.lulzbot.com/cura.

[66] Rankin T.M., Giovinco N.A., Cucher D.J., Watts G., Hurwitz B., Armstrong D.G. (2014). Three-dimensional printing surgical instruments: are we there yet?. J. Surg. Res..

[67] Heikkinen I.T., Kauppinen C., Liu Z., Asikainen S.M., Spoljaric S., Seppälä J.V., Savin H., Pearce J.M. (2018). Chemical compatibility of fused filament fabrication-based 3-D printed components with solutions commonly used in semiconductor wet processing. Addit. Manuf..

[68] Synthetic Nasal Mucus | Kryolan Available online: https://us.kryolan.com/product/synthetic-nasal-mucus (accessed on May 6, 2020).

[69] 3D printing and worker safety [Internet]. [cited 2020 May 15]. Available from: https://www.safetyandhealthmagazine.com/articles/18295-d-printing-and-worker-safety.

[70] Ultraviolet (UV) Radiation Available online: https://www.cancer.org/cancer/cancer-causes/radiation-exposure/uv-radiation.html (accessed on May 6, 2020).

[71] Isopropyl Alcohol SDS. https://www.kenelec.com.au/wp-content/uploads/2019/03/Kenelec-Isopropyl-Alcohol-90-100-SDS.pdf

[72] U.S. Food and Drug Administration (FDA). 2019. FDA. Home Medical Devices Device Advice: Comprehensive Regulatory Assistance Overview of Device Regulation Classify Your Medical Device Class I / II Exemptions. [WWW Document], URL https://www.fda.gov/medical-devices/classify-your-medical-device/class-i-ii-exemptions (accessed 4.22.20).

[73] CFR § 880.6025 - Absorbent tipped applicator. Electronic Code of Federal Regulations (e-CFR) Title 21. Food and Drugs Chapter I. FOOD AND DRUG ADMINISTRATION, DEPARTMENT OF HEALTH AND HUMAN SERVICES Subchapter H. MEDICAL DEVICES Part 880. GENERAL HOSPITAL AND PERSONAL USE DEVICES Subpart G. General Hospital and Personal Use Miscellaneous Devices Section 880.6025. Absorbent tipped applicator. [WWW Document], Legal Information Institute. URL https://www.law.cornell.edu/cfr/text/21/880.6025 (accessed 4.22.20).

[74] D.G. Goodwin, Personal communication. FDA/CDRH/OHT7 (OIR)/DMD May 6, 2020.

[75] CFR – Code of Federal Regulations Title 21 [WWW Document], 2019 URL https://www.accessdata.fda.gov/scripts/cdrh/cfdocs/cfcfr/CFRSearch.cfm?fr=807.20 (accessed 4.22.20).

[76] M.K. Soehnlen, Director of Infectious Disease, Michigan Department of Health and Human Services. Personal communication 5-6-2020.

[77] J.M. Pearce, Expanding the Consumer Bill of Rights for material ingredients. Materials Today 21(3), pp. 197-198. doi: 10.1016/j.mattod.2018.02.002.

[78] John F. Kennedy, Special message to the Congress on protecting the consumer interest, Public papers of the presidents of the United States 93 (1962) 236. Available online: http://www.presidency.ucsb.edu/ws/?pid=9108.

[79] Salmi M., Akmal J.S., Pei E., Wolff J., Jaribion A., Khajavi S.H. (2020). 3D printing in COVID-19: productivity estimation of the most promising open source solutions in emergency situations. Appl. Sci..

[80] Material Safety Data Sheet. Photopolymer Resin. https://shop.prusa3d.com/fotky/SafetySheet_Resin_Tough.pdf (visited 7-28-2020).

[81] M. Perez, M. Block, D. Espalin, R. Winker, T. Hoppe, F. Medina, R. Wicker, August. Sterilization of FDM-manufactured parts. In 23rd Annual International Solid Freeform Fabrication Symposium–An Additive Manufacturing Conference (pp. 285-296), 2012.

[82] Sosnowski E.P., Morrison J. (2017). Sterilization of medical 3D printed plastics: Is H2O2 vapour suitable?. CMBES Proceedings.

[83] N.G. Skrzypczak, N.G. Tanikella, J.M. Pearce, Open Source High-Temperature RepRap for 3-D Printing Heat-Sterilizable PPE and Other Applications. HardwareX 8 (2020) e00130. https://www.preprints.org/manuscript/202005.0479/v1. 10.1016/j.ohx.2020.e00130 PMC7391241 32838090

[84] Zerohealth. 3D-printed nasopharyngeal swab for COVID-19 https://www.thingiverse.com/thing:4259605 (visited 7-28-2020).

[85] Zerohealth. 3D-printed nasopharyngeal swab for COVID-19 v2 flexiblehttps://www.thingiverse.com/thing:4344545 (visited 7-28-2020).

[86] Siderits. Nasal – sample swab mid turbinate TPU https://3dprint.nih.gov/discover/3dpx-014345 (visited 7-28-2020).

[87] D Printed Nasopharyngeal Testing Swab Prototype https://dprint.nih.gov/discover/DPX-014440 (visited 7-28-2020)

[88] Validation Plan: 3D Printed Swab Bridging Study. FINAL 5/6/2020.

[89] United States Food and Drug Administration. FAQs on diagnostic testing for SARS-CoV-2. Retrieved from https://www.fda.gov/medical-devices/emergency-situations-medical-devices/faqs-diagnostic-testing-sars-cov-2, 2020.

